# Diversity of *Pholcus* Spiders (Araneae: Pholcidae) in China’s Lüliang Mountains: An Integrated Morphological and Molecular Approach

**DOI:** 10.3390/insects14040364

**Published:** 2023-04-06

**Authors:** Fang-Yu Zhao, Lan Yang, Quan-Xuan Zou, Abid Ali, Shu-Qiang Li, Zhi-Yuan Yao

**Affiliations:** 1 College of Life Science, Shenyang Normal University, Shenyang 110034, China; 2 Department of Entomology, Faculty of Agriculture, University of Agriculture, Faisalabad 38040, Punjab, Pakistan; 3 Institute of Zoology, Chinese Academy of Sciences, Beijing 100101, China; 4 Liaoning Key Laboratory of Evolution and Biodiversity, Shenyang 110034, China; 5 Liaoning Key Laboratory for Biological Evolution and Agricultural Ecology, Shenyang 110034, China

**Keywords:** biodiversity, molecular species delimitation, morphology, phylogeny, systematics

## Abstract

**Simple Summary:**

*Pholcus* is the most diverse spider genus in Pholcidae, and is widely distributed in the Palaearctic, Indo-Malayan, Afrotropical, and Australasian Regions. Previously, the *Pholcus* spiders have not been recorded from the Lüliang Mountains of North China. We undertook an expedition there for the first time. Phylogenetic analyses of DNA sequence data from four gene fragments (COI, H3, wnt, 28S) suggested that *Pholcus* from the Lüliang Mountains were grouped into nine well-supported clades. We adopted an integrative approach, including morphology and four methods of molecular species delimitation (ABGD, GMYC, bPTP, and BPP), to investigate species boundaries. Such analyses identified the nine clades as nine separate species, of which eight are new to science. All of them belong to the *P. phungiformes* species group.

**Abstract:**

Spiders of the genus *Pholcus* were collected for the first time during an expedition to the Lüliang Mountains in Shanxi Province, North China. Phylogenetic analyses of DNA sequence data from COI, H3, wnt, and 28S genes allowed us to group them into nine well-supported clades. We used morphology and four methods of molecular species delimitation, namely Automatic Barcode Gap Discovery (ABGD), the Generalized Mixed Yule Coalescent (GMYC), Bayesian Poisson Tree Processes (bPTP), and Bayesian Phylogenetics and Phylogeography (BPP), to investigate species boundaries. These integrative taxonomic analyses identified the nine clades as nine distinct species, comprising *Pholcus luya* Peng & Zhang, 2013 and eight other species new to science: *Pholcus jiaocheng* sp. nov., *Pholcus linfen* sp. nov., *Pholcus lishi* sp. nov., *Pholcus luliang* sp. nov., *Pholcus wenshui* sp. nov., *Pholcus xiangfen* sp. nov., *Pholcus xuanzhong* sp. nov., and *Pholcus zhongyang* sp. nov. The species occur in geographic proximity and show many morphological similarities. All of them belong to the *P. phungiformes* species group. The records from the Lüliang Mountains represent the westernmost distribution limit of this species group.

## 1. Introduction

The concept of “biodiversity hotspots” was developed by conservation biologists to highlight areas with exceptional concentrations of endemic species simultaneously experiencing exceptional loss of habitat [1,2]. Although such hotspots are determined mainly by high species richness of vascular plants and vertebrate species, it is assumed that they also harbor correspondingly high diversity of invertebrates [1]. Two such hotspots fall within China’s territories: first, the mountains of Southwest China, and second, portions of what is termed “Indo–Burma” which includes parts of Yunnan, Guangxi, Guangdong, and Hainan [2,3]. Even though North China and Northeast China are not considered to be biodiversity hotspots, this study reinforces the overall impression that an exceptional concentration of endemic species of spiders of the *Pholcus phungiformes* species group occurs in both regions.

Almost all the spiders of the *Pholcus phungiformes* species group described so far are recorded from three mountain ranges: the Yanshan–Taihang Mountains in North China, the Changbai Mountains at the border between Northeast China and North Korea, and the Taebaek Mountains in the Korean Peninsula [4]. Five members of the species group were first recorded in the Yanshan–Taihang Mountains between 1991 and 2000 [5,6,7]. Another 17 new species were added to the inventory of the same mountain range in the next 20 years [4,8,9,10,11,12,13,14,15,16]. We carried out a systematic investigation in the Yanshan–Taihang Mountains in 2021 and collected 29 species, of which 13 species were new to science [17], bringing the total fauna of this species group in the Yanshan–Taihang Mountains to 35 species. With regard to the Changbai Mountains, only 14 species were recorded from 1994 to 2020. Following a wide-ranging expedition into the mountains in 2020, we reported 12 new species and suggested that species diversity in this area might still be underestimated [18,19]. For the Taebaek Mountains, 37 species have been recorded successively from 1978, although several species need to be further studied and illustrated [20]. Taken together, the species group shows a surprisingly high degree of diversity and endemicity. The only exception to its endemicity is *P. phungiformes* Oliger, 1983, which is widely distributed throughout the Russian Far East, stretching from the Maritime Territory to the Sakhalin and Kurile Islands, probably as a result of human transport [21].

**Figure 1:**
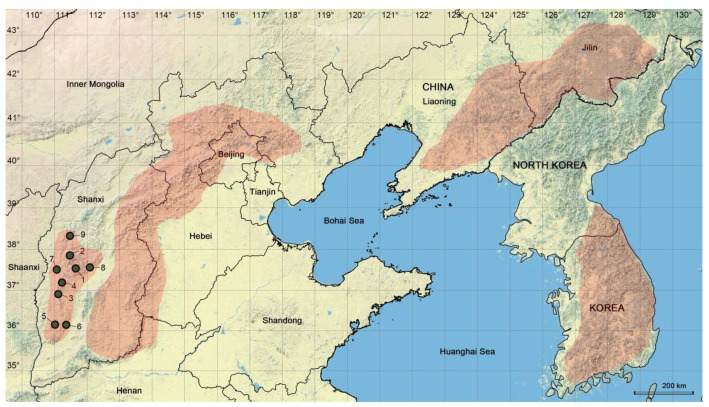
Distribution records of the *Pholcus phungiformes* species group from the Lüliang Mountains, China. Red shadows from left to right indicate the Lüliang Mountains, the Yanshan–Taihang Mountains, the Changbai Mountains, and the Taebaek Mountains, respectively. 1: *P*. *wenshui* sp. nov.; 2: *P*. *jiaocheng* sp. nov.; 3: *P*. *luliang* sp. nov.; 4: *P*. *zhongyang* sp. nov.; 5: *P*. *linfen* sp. nov.; 6: *P*. *xiangfen* sp. nov.; 7: *P*. *lishi* sp. nov.; 8: *P*. *xuanzhong* sp. nov.; 9: *P*. *luya*.

The present study focuses on spiders of the *Pholcus phungiformes* species group in the Lüliang Mountains in the west of North China’s Shanxi Province (Figure 1), an area where spiders of the genus *Pholcus* have not been recorded previously. We searched for them extensively in the mountain range and found that these species were morphologically similar to one another. It is important that species are accurately identified so that it will not lead to misunderstanding and spurious interpretations of biological processes in various domains of the life sciences [22,23,24], and so we adopted an integrative approach in delineating the species, with reference to Crespo et al., Wang et al. and Lu et al. [17,25,26]. Based on the subtle morphological differences and DNA sequence data from both mitochondrial and nuclear genes, we defined their species boundaries. Nine species were identified, of which eight are new to science. Morphology-based species descriptions of these eight new species are given in the latter half of this paper.

## 2. Materials and Methods

### 2.1. Morphological Observation

Specimens were examined and measured with a Leica M205 C stereomicroscope. Left male pedipalps were photographed (unless otherwise indicated in figure legends). Epigynes were photographed before dissection. Vulvae were treated in a 10% warm solution of potassium hydroxide (KOH) to dissolve soft tissues before illustration. Images were captured with a Canon EOS 750D wide zoom digital camera (24.2 megapixels) mounted on the stereomicroscope mentioned above and assembled using Helicon Focus 3.10.3 image stacking software [27]. All measurements are given in millimeters (mm). Leg measurements are shown as: total length (femur, patella, tibia, metatarsus, tarsus). Leg segments were measured on their dorsal side. The specimens studied were preserved in 75% ethanol and deposited in the College of Life Science, Shenyang Normal University (SYNU) in Liaoning, China. Terminology and taxonomic descriptions follow Huber [21] and Yao et al. [18,28]. This published work and the nomenclatural acts it contains have been registered in ZooBank, the online registration system for the ICZN. The LSID for this publication is: urn:lsid:zoobank.org:pub:4CAA98A6-2CF3-44C8-94A5-4B363E23B603. The distribution map was generated with ArcGIS 10.2 (ESRI Incorporated Company, Redlands, CA, USA).

### 2.2. Phylogenetic Analyses

Genomic DNA extraction and amplification were performed as in Yao et al. [29]. We targeted four DNA fragments for sequencing: the mitochondrial gene fragment encoding COI and three nuclear gene fragments encoding H3, wnt and 28S. Two species *Pholcus paralinzhou* Zhang & Zhu, 2009 and *P. taishan* Song & Zhu, 1999 were selected as outgroups. Primers are listed in the Supporting Information (Table S1). DNA sequences were checked and edited with BioEdit 7.2.2 [30]. Bayesian inference (BI) and maximum likelihood (ML) methods were used to reconstruct phylogenetic trees using both COI and a combined dataset. BI analysis was performed with MrBayes 3.2.4 [31]. The GTR + I + G model was used for the concatenated data. Two simultaneous runs of four Monte Carlo Markov chains (MCMCs) with default heating parameters were performed for 4 million generations. Trees were sampled every 1000 generations with the first 25% of sampled trees discarded as burn-in. The results were checked using Tracer 1.6 [32] to ensure stationarity. ML analysis was conducted using RAxML 8.2.9 [33] under a GTRCAT model for all partitions, with 500 rapid bootstrap replicates followed by a thorough maximum likelihood tree search. The phylogenetic results using COI and the concatenated data were used to perform the analyses of molecular species delimitation below.

### 2.3. Molecular Species Delimitation

We applied four methods for molecular species delimitation. The Automatic Barcode Gap Discovery (ABGD) online version examines species delimitation with recursive partitioning using a range of prior intraspecific divergence and relative gap widths, estimating the threshold between intra- and interspecific genetic variation to generate species-level groupings. The ABGD analyses were conducted using both Jukes–Cantor and Kimura 2-P distance matrices with options: Pmin = 0.001, Pmax = 0.1, Steps = 10, X = 1.0, Nb bins = 20 [34].

The Bayesian implementation of the Poisson Tree Processes (bPTP) model tests species boundaries based on phylogenetic trees of individual genes. The bPTP method uses nucleotide substitution information and implements a model assuming phylogenetic tree branch lengths are generated by two classes of Poisson processes (intra- and interspecific branching events). This analysis was conducted on a web server (http://species.h-its.org/ptp/, accessed on 17 January 2023) using individual gene trees. The MCMC was run for 100,000 generations, with a thinning of 100 and burn-in of 0.2 [35].

The Generalized Mixed Yule Coalescent (GMYC) model delimits species from an ultrametric tree of individual genes without prior definitions of species. The GMYC method identifies a time point on the best tree (the tree with the highest likelihood) where the branching rate shifts from speciation to the population coalescent process. This analysis was performed under the single-threshold model using the R 4.2.2 package SPLITS (Species Limits by Threshold Statistics) [36].

Bayesian Phylogenetics and Phylogeography (BPP) is a multilocus coalescent species delimitation analysis, which requires data from multiple genes and pre-defined candidate species [37,38]. The BPP method accommodates species phylogeny as well as lineage sorting due to ancestral polymorphism and estimates the posterior distribution for different species delimitation models. We used BPP to test apparently conflicting results between the analyses mentioned above. Like Leaché and Fujita [39], we conducted four different sets of analyses with different values of *α* and *β*: (i) *Gθ*(1, 10) and *Gτ*(1, 10), assuming large ancestral population sizes and deep divergences between species; (ii) *Gθ*(2, 2000) and *Gτ*(2, 2000), assuming small ancestral populations and shallow divergences; (iii) *Gθ*(1, 10) and *Gτ*(2, 2000), assuming large ancestral populations and shallow divergences; and (iv) *Gθ*(2, 2000) and *Gτ*(1, 10), assuming small ancestral populations and deep divergences. The analyses were performed using the following settings: species delimitation = 1, algorithm = 0, finetune = 5. The reversible-jump MCMC analyses were run for 100,000 generations and sampled every two generations, with 25,000 samples being discarded as burn-in. Each set of *α* and *β* was run at least twice to confirm consistency.

## 3. Results

### 3.1. Morphological Variations

**Figure 2:**
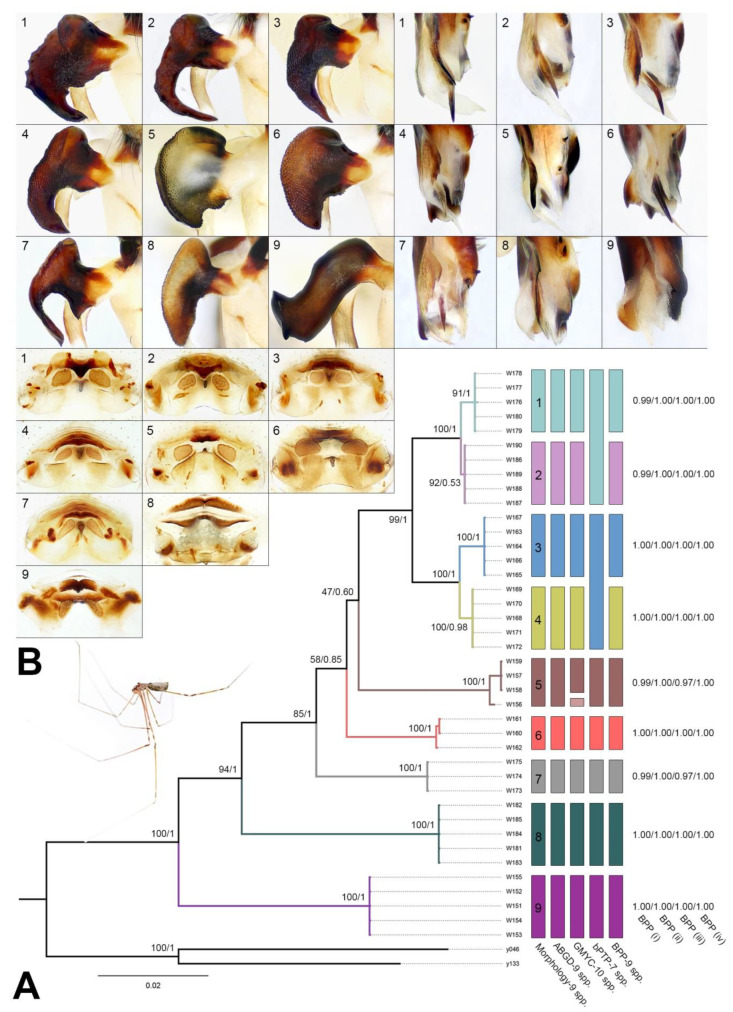
The results of species delimitation analyses: (**A**) Phylogenetic tree inferred from BI analysis, boostrap values/Bayesian posterior probabilities are provided at the nodes (species delimitation methods and posterior probabilities from BPP are presented on the right, different colors of the bars represent the different species); (**B**) Uncus, distal part of procursus, and vulva (more details are provided under the Taxonomic accounts, see below). 1: *P*. *wenshui* sp. nov.; 2: *P*. *jiaocheng* sp. nov.; 3: *P*. *luliang* sp. nov.; 4: *P*. *zhongyang* sp. nov.; 5: *P*. *linfen* sp. nov.; 6: *P*. *xiangfen* sp. nov.; 7: *P*. *lishi* sp. nov.; 8: *P*. *xuanzhong* sp. nov.; 9: *P*. *luya*.

A total of nine populations and 42 specimens were studied. Most morphological variation occurs in the shape of the uncus and the presence or absence of its distal apophysis, the presence or absence of the distal apophyses on the procursus, the shape of the vulval pore plate (Figure 2B), and the presence or absence of the frontal apophysis of the male clypeus. Based on the above characters, the nine populations were identified as nine distinct species. More detailed diagnoses, descriptions, and illustrations are provided under the Taxonomic accounts (see below).

### 3.2. Phylogenetic Relationships

All loci were successfully sequenced for all individuals. A total of 120 sequences of four gene fragments from 40 ingroup members, and four sequences of two gene fragments from two outgroup members were generated. We obtained a concatenated alignment of 2607 bp (COI, 1189 bp; H3, 300 bp; wnt, 292 bp; 28S, 826 bp). The sequences are deposited in the GenBank under accession Nos. OQ706157–706196, OQ719631–719670, OQ719671–719710, OQ719758–719797 (Supporting Information, Table S2). Separate analyses of individual gene COI and concatenated data found compatible topologies (Supporting Information, Figure S1). The BI and ML analyses of the concatenated data supported the same topology. Figure 2A presents the tree from the BI analysis. The *Pholcus phungiformes* species group was clearly divided into nine well-supported major clades and these clades consisted of samples from a single population, therefore, we defined the nine clades as nine candidate species.

### 3.3. Species Delimitation

The ABGD analysis identified nine provisional species using both Jukes–Cantor and Kimura 2-P distance matrices, and the result was fairly consistent with morphology (Figure 2A). The ML tree of COI was used to conduct the GMYC and bPTP analyses due to the same topology of the ML and BI trees. The GMYC analysis identified 10 species, with *Pholcus linfen* sp. nov. (as identified from morphology and ABGD) divided into two species (Figure 2A: number 5, brown and light brown boxes; Supporting Information, Figure S2B). The results from the bPTP were largely consistent with those from morphology and ABGD, except that *Pholcus wenshui* sp. nov. and *Pholcus jiaocheng* sp. nov. were recognized as a single species, while *Pholcus luliang* sp. nov. and *Pholcus zhongyang* sp. nov. were recognized as another species (Figure 2A: numbers 1 and 2, 3 and 4; Supporting Information, Figure S2A). The BPP analysis requires pre-defined species and phylogenetic relationships of these species. Based on the results of morphology, ABGD, and GMYC, as well as the ML tree of the concatenated data, we used BPP to validate the nine species. The BPP analyses found very high probabilities of speciation events for all of the nodes tested using all four prior combinations. In particular, four prior combinations produced speciation probabilities of one for most of the nodes (Figure 2A: BPP i–iv). These results were also consistent across multiple runs.

## 4. Discussion

### 4.1. How Many Members of the P. phungiformes Species Group Are There in the Lüliang Mountains?

Considering all evidence, we conclude that there are nine species from the Lüliang Mountains. The phylogenetic tree derived from the concatenated data clearly divided the samples into nine deeply divergent clades (Figure 2A). Moreover, the ABGD analysis supports speciation events among the nine species and the result is fairly consistent with morphology.

Although the GMYC analysis divided the species *P. linfen* sp. nov. (Figure 2A: number 5) into two species, this delimitation result is unreasonable because all four samples (W156–W159) are from the same population. In addition, a single speciation event for *P. linfen* sp. nov. is well supported by ABGD, bPTP, and BPP analyses. The morphological characters of specimens from the same population are also consistent, e.g., the prolateral membranous process of the procursus with a curved sclerotized apophysis, the procursus with a slightly sclerotized ventro–distal apophysis and without spine-shaped distal apophyses, and the nearly trapezoidal vulval pore plates.

The bPTP collapsed two species pairs each to a single species: *P. wenshui* sp. nov. and *P. jiaocheng* sp. nov. (Figure 2A: numbers 1 and 2); *P. luliang* sp. nov. and *P. zhongyang* sp. nov. (Figure 2A: numbers 3 and 4). Nevertheless, the other three molecular delimitation results clearly support their status as separate species. Furthermore, *P*. *wenshui* sp. nov. can be distinguished morphologically from *P*. *jiaocheng* sp. nov. by the prolateral membranous process of the procursus with a strongly sclerotized edge, the procursus with a spine-shaped distal apophysis, the uncus with a wide distal apophysis and a sawtoothed edge, and the epigynal plate strongly curved in the ventral view. *P. luliang* sp. nov. can be distinguished morphologically from *P. zhongyang* sp. nov. by the procursus with a spine-shaped distal apophysis and without sclerotized ventro–distal apophyses, the uncus with a slightly curved distal apophysis, and the nearly round vulval pore plates.

Finally, the BPP analyses unambiguously support the speciation events among those nine clades, with the posterior probabilities of most of the nodes are one in four prior combinations. For the two species pairs collapsed by the bPTP, the possibility of *P. wenshui* sp. nov. and *P. jiaocheng* sp. nov. recognized as a single species lies in BPP ii and iv, but their posterior probabilities are very low, 0.007 and 0.0007, respectively. Similarly, the possibility of *P. luliang* sp. nov. and *P. zhongyang* sp. nov. being delimited to one species was found only in BPP iv, and the posterior probability was only 0.001.

We employed morphology and four commonly used molecular methods for the *P. phungiformes* species group, and produced fairly consistent results, except for slight deviations arising from GMYC and bPTP analyses. On this basis, we can assert that combining morphological with molecular data allows for rapid and accurate assessment of species richness and therefore such an approach can be considered as an essential part of the conservationist’s toolkit. It should however be noted that several molecular-based methods of species delimitation that have been proposed and often applied (e.g., Dincă et al.; Dumas et al.; Li et al. [40,41,42]) have yielded different conclusions. For this reason, the strengths and weakness of each of these methods may still need to be further explored and evaluated.

### 4.2. Comparison of Species Diversity within the P. phungiformes Species Group

Based on the samples collected from the nine populations in the Lüliang Mountains, each is a distinct species. This degree of diversity is comparable proportionally with that found in the Yanshan–Taihang Mountains and the Changbai Mountains. The Yanshan–Taihang Mountains are almost four times the size of the Lüliang Mountains, and they harbor 35 species, almost four times the diversity of the latter [17]. Similarly, both the total area and diversity of the Changbai Mountains [18] are approximately three times those of the Lüliang Mountains. The Taebaek Mountains of the Korean Peninsula are an exception. Their area is just over twice that of the Lüliang Mountains, but with 37 species recorded there [20], its diversity is more than four times that of the latter. Such disparity suggests there may well be species from the group that are yet to be discovered in the three Chinese mountain ranges. This belief is rooted in the fact that all four mountain ranges have similar landforms and ecological niches occupied by the spiders, e.g., rock walls in montane mixed forests. Furthermore, the Lüliang Mountains and the Yanshan–Taihang Mountains are located within the same latitudinal belt as the Taebaek Mountains (35° to 40° N).

The Lüliang Mountains probably represent the westernmost distribution limit of spiders of the *P. phungiformes* species group. None of this group could be found when we sampled specimens of *Pholcus* in the adjacent Shaanxi Province intensively and extensively in 2013, 2016, and 2019, covering all prefectures throughout the province. They were again absent in the collections from our 2022 expedition to the Qinling Mountains whose range extends from Shaanxi Province to the western part of Henan Province.

### 4.3. Geographic Proximity and Morphological Similarities within the P. phungiformes Species Group from the Lüliang Mountains

Of the nine species from the Lüliang Mountains, eight species are new to science. More significantly, they are in close geographic proximity. For instance, the sister species *P. wenshui* sp. nov. and *P. jiaocheng* sp. nov. (numbers 1 and 2 in Figure 1) were 49 km apart. Another two sister species, *P. luliang* sp. nov. and *P. zhongyang* sp. nov. (numbers 3 and 4), were found only 42 km apart. The species with the nearest distance in geography are *P. linfen* sp. nov. and *P. xiangfen* sp. nov. (numbers 5 and 6). They were found only 39 km apart. Additionally, *P. lishi* sp. nov. (number 7) was found approximately 49 km away from the nearest species *P. zhongyang* sp. nov. (number 4), and *P. xuanzhong* sp. nov. (number 8) was found 48 km away from the nearest *P. wenshui* sp. nov. (number 1).

All the new species are similar to each other in their morphology. For instance, in males, the procursus is highly complex distally but includes the same three structures: a prolateral membranous process, a dorsal membranous lamella, and dorsal spines (e.g., arrows 1, 3 in Figure 9C, arrows in Figure 9D). Six species, excluding *P. linfen* sp. nov. and *P. xuanzhong* sp. nov., possess a spine-shaped or sclerotized distal apophysis on their procursus (e.g., arrow 2 in Figure 9C). Five species, namely *P*. *wenshui* sp. nov., *P*. *jiaocheng* sp. nov., *P*. *luliang* sp. nov., *P*. *zhongyang* sp. nov., and *P*. *lishi* sp. nov., possess a curved distal apophysis on the uncus (e.g., arrow 1 in Figure 10C); the uncus of the three other species is nearly semi-circular or elliptic (e.g., Figure 6C). Five species, namely *P*. *wenshui* sp. nov., *P*. *jiaocheng* sp. nov., *P*. *luliang* sp. nov., *P*. *zhongyang* sp. nov., and *P. linfen* sp. nov., have a small frontal apophysis on their clypeus. In females, the epigynal plate of all the new species except for *P. xuanzhong* sp. nov. is posteriorly curved.

Such geographic proximity, as well as the morphological similarities, suggests that this group might have undergone a recent radiation. Furthermore, all of these new species are found only on rock walls from the Lüliang Mountains. This group may have become specialized in living on rock walls, although we have not detected any particular adaptive or physiological traits. It is uncertain whether such specialization could have hampered their dispersal and gene interchange among populations between different rock walls in the mountainous region, thereby paving the way for geographic isolation and species radiation. These questions are challenging subjects for further investigation into this species group.

## 5. Taxonomic Accounts

Family Pholcidae C.L. Koch, 1850

Subfamily Pholcinae C.L. Koch, 1850

Genus *Pholcus* Walckenaer, 1805

Type species: *Aranea phalangioides* Fuesslin, 1775

*Pholcus phungiformes* species group

**Figure 3:**
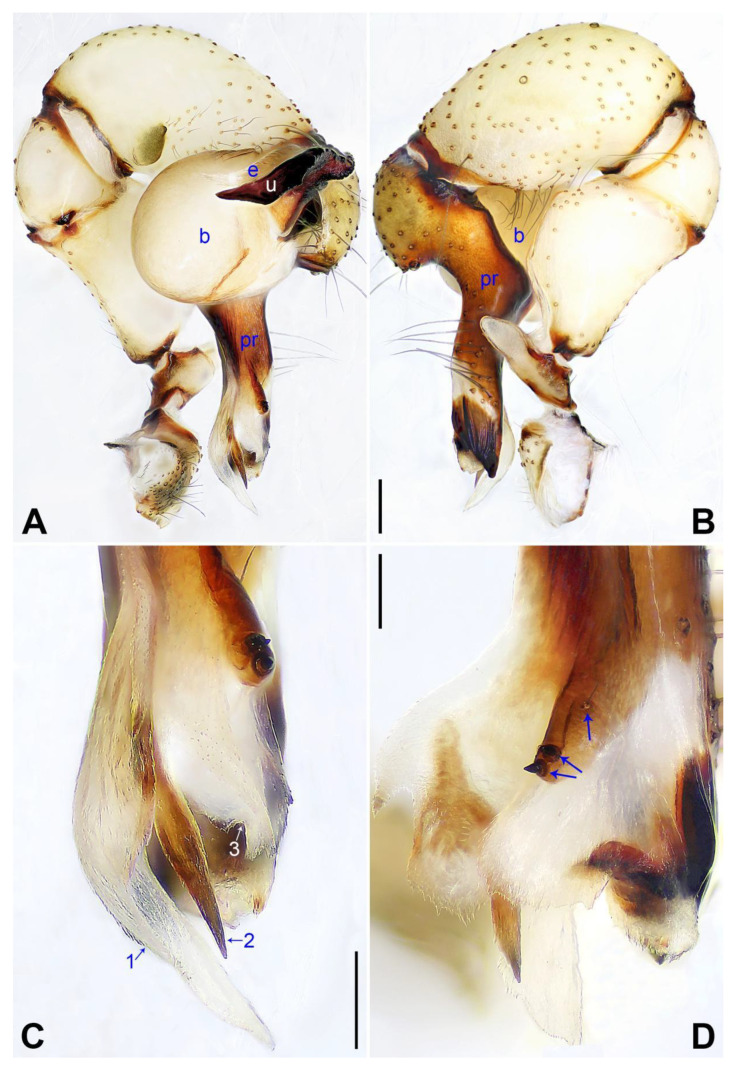
*Pholcus jiaocheng* sp. nov., holotype male: (**A**) pedipalp, prolateral view; (**B**) pedipalp, retrolateral view; (**C**) distal part of procursus, prolateral view (arrow 1 points at prolateral membranous process, arrow 2 points at pointed distal apophysis, arrow 3 points at dorsal membranous lamella); (**D**) distal part of procursus, dorsal view (arrows point at dorsal spines). b = bulb, e = embolus, pr = procursus, u = uncus. Scale bars: (**A**,**B**) = 0.20 mm; (**C**,**D**) = 0.10 mm.

These species below are assigned to the *phungiformes* group by the following combination of characters: male chelicerae with frontal apophyses (e.g., arrow fa in Figure 3D), male pedipalpal tibia with a prolatero–ventral projection (e.g., Figure 2A), uncus with a “pseudo-appendix” (e.g., arrow 2 in Figure 3C), and epigyne with a knob (Figure 3A).

***Pholcus jiaocheng* Zhao, Li & Yao, sp. nov.**

**Figure 4:**
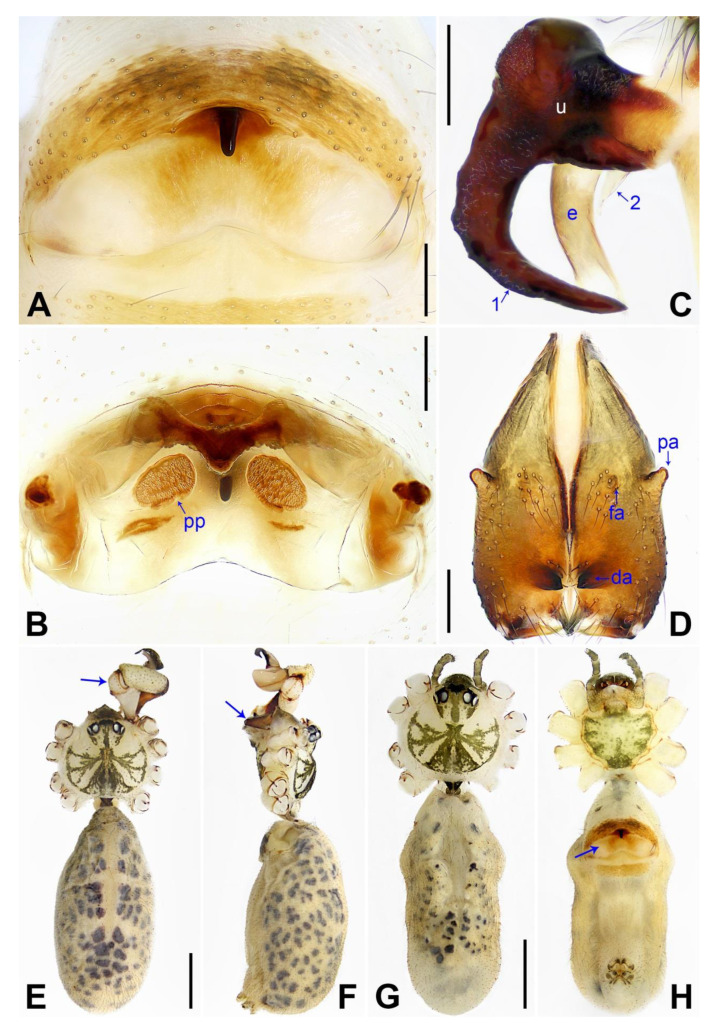
*Pholcus jiaocheng* sp. nov., holotype male (**C**–**F**) and paratype female (**A**,**B**,**G**,**H**): (**A**) epigyne, ventral view; (**B**) vulva, dorsal view; (**C**) bulbal apophyses, prolateral view (arrow 1 points at slender distal apophysis, arrow 2 points at “pseudo-appendix”); (**D**) chelicerae, frontal view; (**E**), (**G**) habitus, dorsal view (arrow points at pedipalp); (**F**) habitus, lateral view (arrow points at chelicerae); (**H**) habitus, ventral view (arrow points at epigyne). da = distal apophysis, e = embolus, fa = frontal apophysis, pa = proximo–lateral apophysis, pp = pore plate, u = uncus. Scale bars: (**A**–**D**) = 0.20 mm; (**E**–**H**) = 1.00 mm.

LSID: urn:lsid:zoobank.org:act:B35A9380-213A-424B-AFDE-FE18A9B4B6F3 

(Figure 3 and Figure 4).

**Holotype:** ♂ (SYNU-Ar00255), China, Shanxi, Lüliang, Jiaocheng County, Pangquangou Town, near Pangquangou Nature Reserve, Badaogou Scenic Spot (37°50.97′ N, 111°28.23′ E, 1755 m), 6 August 2022, Zhi-Yuan Yao, Lan Yang & Lu-Dan Zhang leg.

**Paratypes:** 2♂ (SYNU-Ar00256, Ar00257), 2♀ (SYNU-Ar00258, Ar00259), same data as holotype.

**Etymology:** The specific name refers to the type locality and is a noun in apposition.

**Diagnosis:** The species resembles *P. wenshui* sp. nov. in having similar male chelicerae and vulva (Figure 12B,D), but can be distinguished by a prolateral membranous process of procursus with a slightly sclerotized edge (arrow 1 in Figure 3C; strongly sclerotized edge in *P. wenshui* sp. nov., arrow 1 in Figure 11C), by a procursus with a slightly sclerotized, pointed distal apophysis (arrow 2 in Figure 3C; spine-shaped distal apophysis in *P. wenshui* sp. nov., arrow 2 in Figure 11C), by an uncus with a slender distal apophysis (arrow1 in Figure 4C; wide distal apophysis and sawtoothed edge in *P. wenshui* sp. nov., arrow 1 in Figure 12C), and by an epigynal plate slightly curved in ventral view (Figure 4A; strongly curved in *P. wenshui* sp. nov., Figure 12A).

**Description of holotype:** Male (SYNU-Ar00255). Total length 5.39 (5.60 with clypeus), carapace 1.54 long, 1.84 wide, opisthosoma 3.85 long, 2.06 wide. Leg I: 38.59 (9.85, 0.68, 9.74, 15.77, 2.55), leg II: 28.07 (7.76, 0.66, 7.05, 10.96, 1.64), leg III: 19.56 (5.71, 0.65, 4.81, 7.12, 1.27), leg IV: 25.54 (7.44, 0.64, 6.35, 9.55, 1.56); tibia I L/d: 65. Eye interdistances and diameters: PME–PME 0.28, PME 0.14, PME–ALE 0.04, AME–AME 0.06, AME 0.11. Sternum width/length: 1.30/1.05. Habitus as in Figure 4E,F. Carapace yellowish, with brown radiating marks and marginal brown bands; ocular area yellowish, with median and lateral brown bands; clypeus and sternum yellowish, with brown marks. Legs yellowish, but dark brown on patellae and whitish on distal parts of femora and tibiae, with darker rings on subdistal parts of femora and proximal and subdistal parts of tibiae. Opisthosoma yellowish, with dorsal and lateral spots. Clypeus with small frontal apophysis (Figure 4E). Chelicerae (Figure 4D) with pair of proximo–lateral apophyses, pair of distal apophyses with two teeth each, and pair of frontal apophyses. Pedipalp as in Figure 3A,B; trochanter with long (longer than wide), retrolaterally strongly bulged ventral apophysis; femur with small retrolatero–proximal apophysis and indistinct ventral protuberance; tibia with prolatero–ventral projection; procursus simple proximally but complex distally, with curved prolateral membranous process (arrow 1 in Figure 3C), slightly sclerotized, pointed distal apophysis (arrow 2 in Figure 3C), dorsal membranous lamella (arrow 3 in Figure 3C), and two strong and one slender dorsal spines (arrows in Figure 3D); uncus with slender, curved distal apophysis and scales (arrow1 in Figure 4C); “pseudo-appendix” semi-transparent (arrow 2 in Figure 4C); embolus weakly sclerotized, with some transparent distal projections (Figure 4C). Retrolateral trichobothrium of tibia I at 6% proximally; legs with short vertical setae on tibiae, metatarsi, and tarsi; tarsus I with 41 distinct pseudosegments.

**Description of paratype:** Female (SYNU-Ar00258). Similar to male, habitus as in Figure 4G,H. Total length 4.80 (4.95 with clypeus), carapace 1.48 long, 1.65 wide, opisthosoma 3.32 long, 1.48 wide; tibia I: 6.80; tibia I L/d: 52. Eye interdistances and diameters: PME–PME 0.23, PME 0.15, PME–ALE 0.05, AME–AME 0.06, AME 0.08. Sternum width/length: 1.11/0.89. Clypeus brown, without frontal apophysis. Epigyne (Figure 4A) postero–medially strongly curved, with median brown marks and knob. Vulva (Figure 4B) with M-shaped, sclerotized anterior arch, pair of nearly elliptic pore plates, and pair of indistinct posterior sclerites.

**Variation:** Tibia I in two paratype males (SYNU-Ar00256, SYNU-Ar00257): 10.06, 10.25. Tibia I in another paratype female (SYNU-Ar00259): 6.65.

**Natural history:** The species was found on rock walls.

**Distribution:** China (Shanxi, type locality; Figure 1).

***Pholcus linfen* Zhao, Li & Yao, sp. nov.**

**Figure 5:**
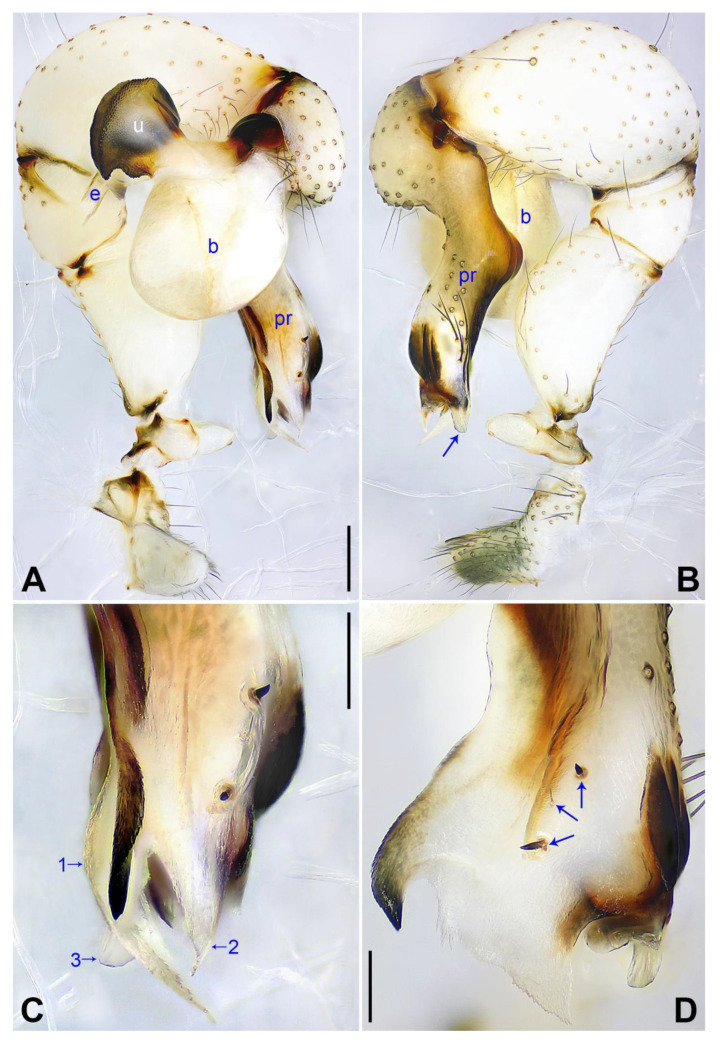
*Pholcus linfen* sp. nov., holotype male: (**A**) pedipalp, prolateral view; (**B**) pedipalp, retrolateral view (arrow points at ventro–distal apophysis); (**C**) distal part of procursus, prolateral view (arrow 1 points at prolateral membranous process, arrow 2 points at dorsal membranous lamella, arrow 3 points at ventro–distal apophysis); (**D**) distal part of procursus, dorsal view (arrows point at dorsal spines). B = bulb, e = embolus, pr = procursus, u = uncus. Scale bars: (**A**,**B**) = 0.20 mm; (**C**,**D**) = 0.10 mm.

**Figure 6:**
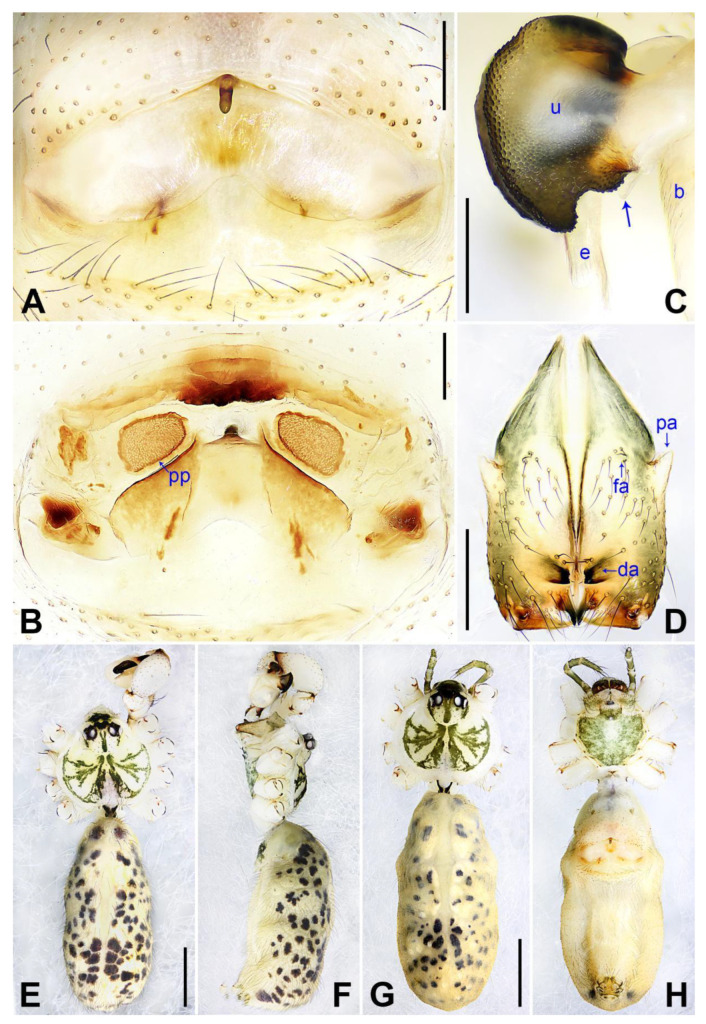
*Pholcus linfen* sp. nov., holotype male (**C**–**F**) and paratype female (**A**,**B**,**G**,**H**): (**A**) epigyne, ventral view; (**B**) vulva, dorsal view; (**C**) bulbal apophyses, prolateral view (arrow points at “pseudo-appendix”); (**D**) chelicerae, frontal view; (**E**), (**G**) habitus, dorsal view; (**F**) habitus, lateral view; (**H**) habitus, ventral view. da = distal apophysis, e = embolus, fa = frontal apophysis, pa = proximo–lateral apophysis, pp = pore plate, u = uncus. Scale bars: (**A**–**D**) = 0.20 mm; (**E**–**H**) = 1.00 mm.

LSID: urn:lsid:zoobank.org:act:057C81CA-8FF6-48CB-B07A-35FC81EEDACF

(Figure 5 and Figure 6).

**Holotype.** ♂ (SYNU-Ar00260), China, Shanxi, Linfen, Ji County, Taitou Town, Wangjiahe Village, roadside of G309 (36°8.87′ N, 111°0.58′ E, 1292 m), 2 August 2022, Zhi-Yuan Yao, Lan Yang & Lu-Dan Zhang leg.

**Paratypes:** 3♂ (SYNU-Ar00261–Ar00263), 4♀ (SYNU-Ar00264–Ar00267), same data as holotype.

**Etymology:** The specific name refers to the type locality and is a noun in apposition.

**Diagnosis:** The species resembles *P. xiangfen* sp. nov. in having similar male chelicerae, uncus and epigyne (Figure 14A,C,D) but can be distinguished by a prolateral membranous process of the procursus with a curved sclerotized apophysis (arrow 1 in Figure 5C; absent in *P. xiangfen* sp. nov., arrow 1 in Figure 13C), by a procursus without spine-shaped distal apophysis (Figure 5C; present in *P. xiangfen* sp. nov., arrow 2 in Figure 13C), by a procursus with a slightly sclerotized ventro–distal apophysis (arrow in Figure 5B, arrow 3 in Figure 5C; with sclerotized ventro–subdistal and ventro–distal apophyses in *P. xiangfen* sp. nov., arrows 4, 5 in Figure 13C), by nearly trapezoidal vulval pore plates (Figure 6B; nearly semi-circular in *P. xiangfen* sp. nov., Figure 14B), and by a male clypeus with a small frontal apophysis (Figure 6E; absent in *P. xiangfen* sp. nov., Figure 14E).

**Description of holotype:** Male (SYNU-Ar00260). Total length 4.90 (5.12 with clypeus), carapace 1.50 long, 1.66 wide, opisthosoma 3.40 long, 1.60 wide. Leg I: 39.59 (9.94, 0.73, 10.06, 16.22, 2.64), leg II: 27.83 (7.84, 0.69, 6.80, 10.90, 1.60), leg III: 18.84 (5.60, 0.66, 4.35, 7.08, 1.15), leg IV: 25.15 (7.41, 0.66, 6.05, 9.50, 1.53); tibia I L/d: 67. Eye interdistances and diameters: PME–PME 0.22, PME 0.20, PME–ALE 0.05, AME–AME 0.06, AME 0.09. Sternum width/length: 1.16/0.98. Habitus as in Figure 6E,F. Carapace yellowish, with brown radiating marks and marginal brown bands; ocular area yellowish, with median and lateral brown bands; clypeus and sternum yellowish, with brown marks. Legs yellowish, but dark brown on patellae and whitish on distal parts of femora and tibiae, with darker rings on subdistal parts of femora and proximal and subdistal parts of tibiae. Opisthosoma yellowish, with dorsal and lateral spots. Clypeus with small frontal apophysis (Figure 6E). Chelicerae (Figure 6D) with pair of proximo–lateral apophyses, pair of distal apophyses with two teeth each, and pair of frontal apophyses. Pedipalp as in Figure 5A,B; trochanter with long (longer than wide), retrolaterally strongly bulged ventral apophysis; femur with small retrolatero–proximal apophysis and indistinct ventral protuberance; tibia with prolatero–ventral projection; procursus simple proximally but complex distally, with curved prolateral membranous process with curved sclerotized apophysis (arrow 1 in Figure 5C), dorsal membranous lamella (arrow 2 in Figure 5C), slightly sclerotized ventro–distal apophysis (arrow in Figure 5B, arrow 3 in Figure 5C), and two strong and one slender dorsal spines (arrows in Figure 5D); uncus semi-circular, with scaly edge (Figure 6C); “pseudo-appendix” semi-transparent (arrow in Figure 6C); embolus weakly sclerotized, with some transparent distal projections (Figure 6C). Retrolateral trichobothrium of tibia I at 5% proximally; legs with short vertical setae on tibiae, metatarsi, and tarsi; tarsus I with 33 distinct pseudosegments.

**Description of paratype:** Female (SYNU-Ar00264). Similar to male, habitus as in Figure 6G,H. Total length 4.79 (4.87 with clypeus), carapace 1.35 long, 1.65 wide, opisthosoma 3.44 long, 1.60 wide; tibia I: 6.92; tibia I L/d: 49. Eye interdistances and diameters: PME–PME 0.16, PME 0.14, PME–ALE 0.06, AME–AME 0.05, AME 0.09. Sternum width/length: 1.04/0.92. Clypeus brown, without frontal apophysis. Epigyne (Figure 6A) postero–medially strongly curved, with median and lateral brown marks and knob. Vulva (Figure 6B) with curved, medially sclerotized anterior arch, pair of nearly trapezoidal pore plates, and pair of triangular median sclerites.

**Variation:** Tibia I in one paratype male (SYNU-Ar00261): 11.21 (leg I lost in SYNU-Ar00262, Ar00263). Tibia I in the other two paratype females (SYNU-Ar00265, Ar00266): 7.76, 8.01 (leg I lost in SYNU-Ar00267).

**Natural history:** The species was found on rock walls.

**Distribution:** China (Shanxi, type locality; Figure 1).

***Pholcus lishi* Zhao, Li & Yao, sp. nov.**

**Figure 7:**
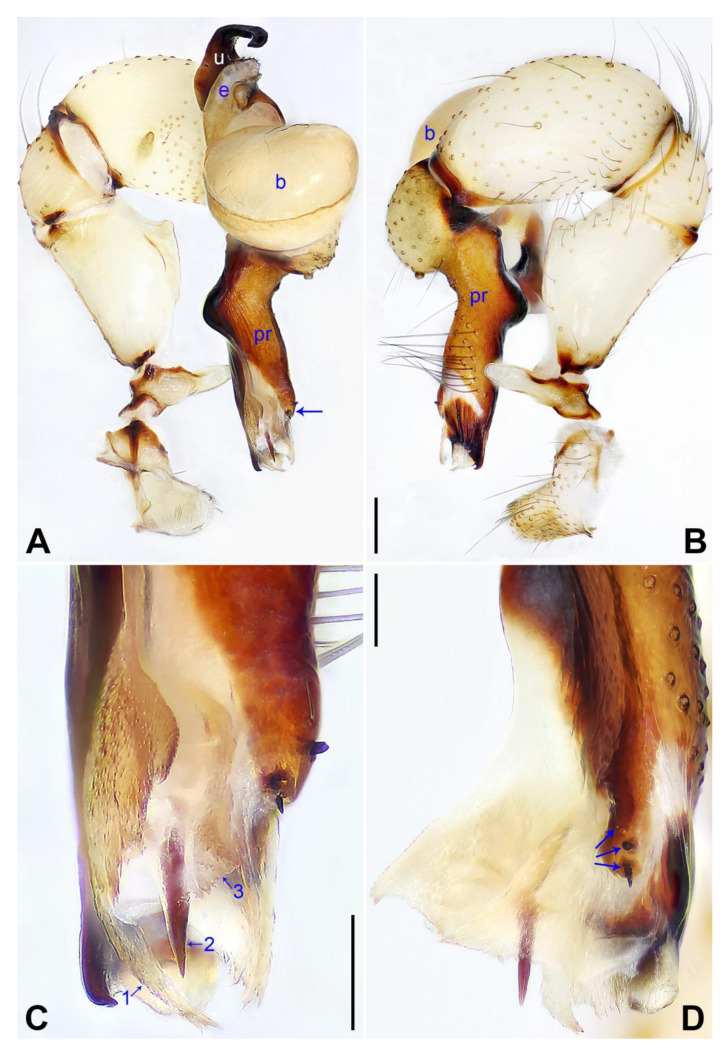
*Pholcus lishi* sp. nov., holotype male: (**A**) pedipalp, prolateral view (arrow points at strongly protruding subdisto–dorsal part); (**B**) pedipalp, retrolateral view; (**C**) distal part of procursus, prolateral view (arrow 1 points at narrow prolateral membranous process, arrow 2 points at spine-shaped distal apophysis, arrow 3 points at indistinct dorsal membranous lamella); (**D**) distal part of procursus, dorsal view (arrows point at dorsal spines). b = bulb, e = embolus, pr = procursus, u = uncus. Scale bars: (**A**,**B**) = 0.20 mm; (**C**,**D**) = 0.10 mm.

**Figure 8:**
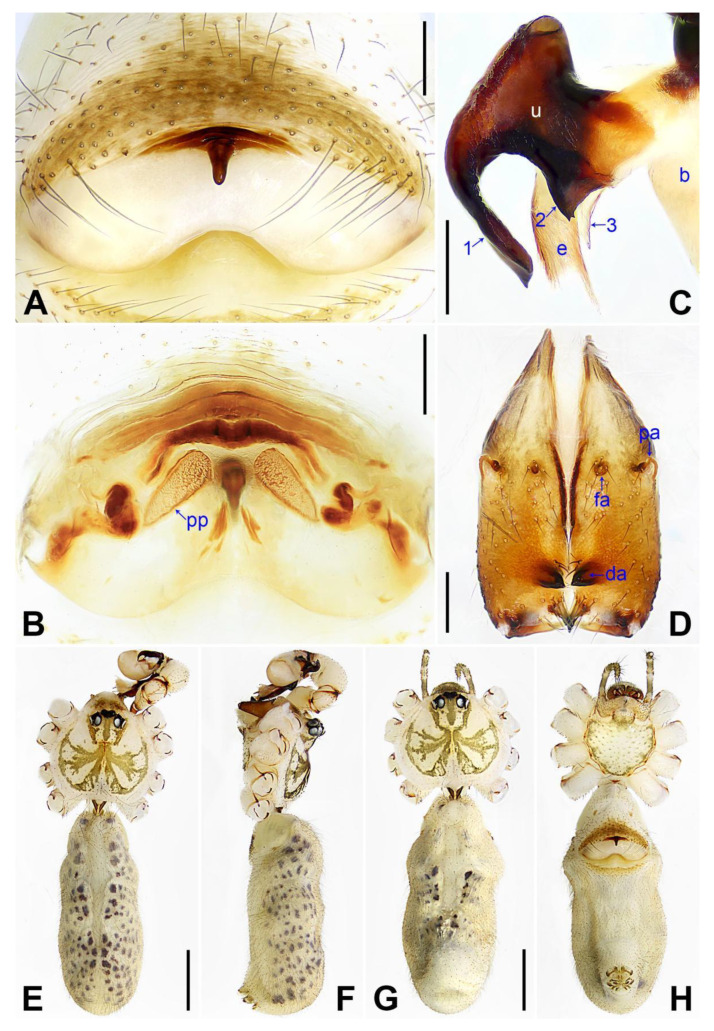
*Pholcus lishi* sp. nov., holotype male (**C**–**F**) and paratype female (**A**,**B**,**G**,**H**): (**A**) epigyne, ventral view; (**B**) vulva, dorsal view; (**C**) bulbal apophyses, prolateral view (arrow 1 points at curved distal apophysis, arrow 2 points at angular proximal apophysis, arrow 3 points at “pseudo-appendix”); (**D**) chelicerae, frontal view; (**E**), (**G**) habitus, dorsal view; (**F**) habitus, lateral view; (**H**) habitus, ventral view. da = distal apophysis, e = embolus, fa = frontal apophysis, pa = proximo–lateral apophysis, pp = pore plate, u = uncus. Scale bars: (**A**–**D**) = 0.20 mm; (**E**–**H**) = 1.00 mm.

LSID: urn:lsid:zoobank.org:act:8F8C4D58-9765-4471-AD6F-CA29C5EF41AC

(Figure 7 and Figure 8).

**Holotype:** ♂ (SYNU-Ar00268), China, Shanxi, Lüliang, Lishi District, Wuya Mountain, near Anguo Temple, roadside of Y004 (37°30.17′ N, 111°4.28’ E, 908 m), 5 August 2022, Zhi-Yuan Yao, Lan Yang & Lu-Dan Zhang leg.

**Paratype:** 1♀ (SYNU-Ar00269), same data as holotype.

**Etymology:** The specific name refers to the type locality and is a noun in apposition.

**Diagnosis:** The species resembles *P. jiaocheng* sp. nov. in having similar male chelicerae and epygine (Figure 4A,D) but can be distinguished by a procursus with a small, narrow prolateral membranous process (arrow 1 in Figure 7C; large and wide in *P. jiaocheng* sp. nov., arrow 1 in Figure 3C), by procursus subdisto–dorsally strongly protruding (arrow in Figure 7A; not protruding in *P. jiaocheng* sp. nov., Figure 3A), by an uncus with an angular proximal apophysis (arrow 2 in Figure 8C; absent in *P. jiaocheng* sp. nov., Figure 4C), by vulval pore plates which are long, anteriorly wide, and posteriorly narrow (Figure 8B; nearly elliptic in *P. jiaocheng* sp. nov., Figure 4B), and by a male clypeus without a small frontal apophysis (Figure 8E; present in *P. jiaocheng* sp. nov., Figure 4E).

**Description of holotype:** Male (SYNU-Ar00268). Total length 4.99 (5.35 with clypeus), carapace 1.44 long, 1.85 wide, opisthosoma 3.55 long, 1.48 wide. Leg I: 40.38 (10.26, 0.79, 10.32, 16.54, 2.47), leg II: 28.57 (7.95, 0.75, 7.18, 11.09, 1.60), leg III: 19.82 (5.71, 0.61, 4.74, 7.56, 1.20), leg IV: 26.51 (7.82, 0.66, 6.70, 9.95, 1.38); tibia I L/d: 69. Eye interdistances and diameters: PME–PME 0.23, PME 0.14, PME–ALE 0.05, AME–AME 0.05, AME 0.09. Sternum width/length: 1.19/1.04. Habitus as in Figure 8E,F. Carapace yellowish, with brown radiating marks and marginal brown bands; ocular area yellowish, with median and lateral brown bands; clypeus yellowish, with brown marks; sternum yellowish, with narrow, marginal brown marks. Legs yellowish, but dark brown on patellae and whitish on distal parts of femora and tibiae, with darker rings on subdistal parts of femora and proximal and subdistal parts of tibiae. Opisthosoma yellowish, with dorsal and lateral spots. Chelicerae (Figure 8D) with pair of proximo–lateral apophyses, pair of distal apophyses with two teeth each, and pair of frontal apophyses. Pedipalp as in Figure 7A,B; trochanter with long (longer than wide), retrolaterally strongly bulged ventral apophysis; femur with small retrolatero–proximal apophysis and distinct ventral protuberance; tibia with prolatero–ventral projection; procursus simple proximally but complex distally, with narrow, curved prolateral membranous process (arrow 1 in Figure 7C), spine-shaped distal apophysis (arrow 2 in Figure 7C), indistinct dorsal membranous lamella (arrow 3 in Figure 7C), and two strong and one slender dorsal spines (arrows in Figure 7D); uncus with narrow curved distal apophysis (arrow 1 in Figure 8C), angular proximal apophysis (arrow 2 in Figure 8C) and scales; “pseudo-appendix” semi-transparent (arrow 3 in Figure 8C); embolus weakly sclerotized, with some transparent distal projections (Figure 8C). Retrolateral trichobothrium of tibia I at 2% proximally; legs with short vertical setae on tibiae, metatarsi, and tarsi; tarsus I with 33 distinct pseudosegments.

**Description of paratype:** Female (SYNU-Ar00269). Similar to male, habitus as in Figure 8G,H. Total length 5.26 (5.45 with clypeus), carapace 1.62 long, 1.92 wide, opisthosoma 3.64 long, 1.56 wide; tibia I: 8.15; tibia I L/d: 45. Eye interdistances and diameters: PME–PME 0.19, PME 0.15, PME–ALE 0.06, AME–AME 0.07, AME 0.09. Sternum width/length: 1.17/0.91. Clypeus brown. Epigyne (Figure 8A) postero–medially strongly curved, with knob. Vulva (Figure 8B) with curved, posteriorly sclerotized anterior arch and pair of long, anteriorly wide and posteriorly narrow pore plates.

**Natural history:** The species was found on rock walls.

**Distribution:** China (Shanxi, type locality; Figure 1).

***Pholcus luliang* Zhao, Li & Yao, sp. nov.**

**Figure 9:**
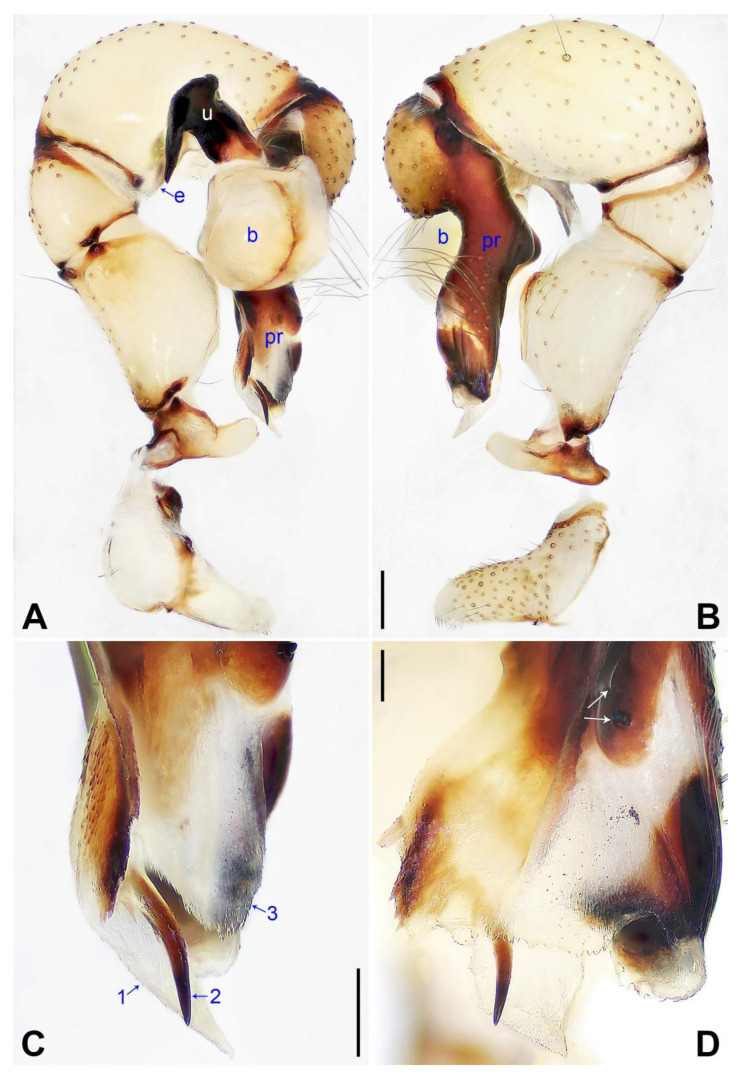
*Pholcus luliang* sp. nov., holotype male: (**A**) pedipalp, prolateral view; (**B**) pedipalp, retrolateral view; (**C**) distal part of procursus, prolateral view (arrow 1 pointed at prolateral membranous process, arrow 2 pointed at spine-shaped distal apophysis, arrow 3 pointed at dorsal membranous lamella); (**D**) distal part of procursus, dorsal view (arrows point at dorsal spines). b = bulb, e = embolus, pr = procursus, u = uncus. Scale bars: (**A**,**B**) = 0.20 mm; (**C**,**D**) = 0.10 mm.

**Figure 10:**
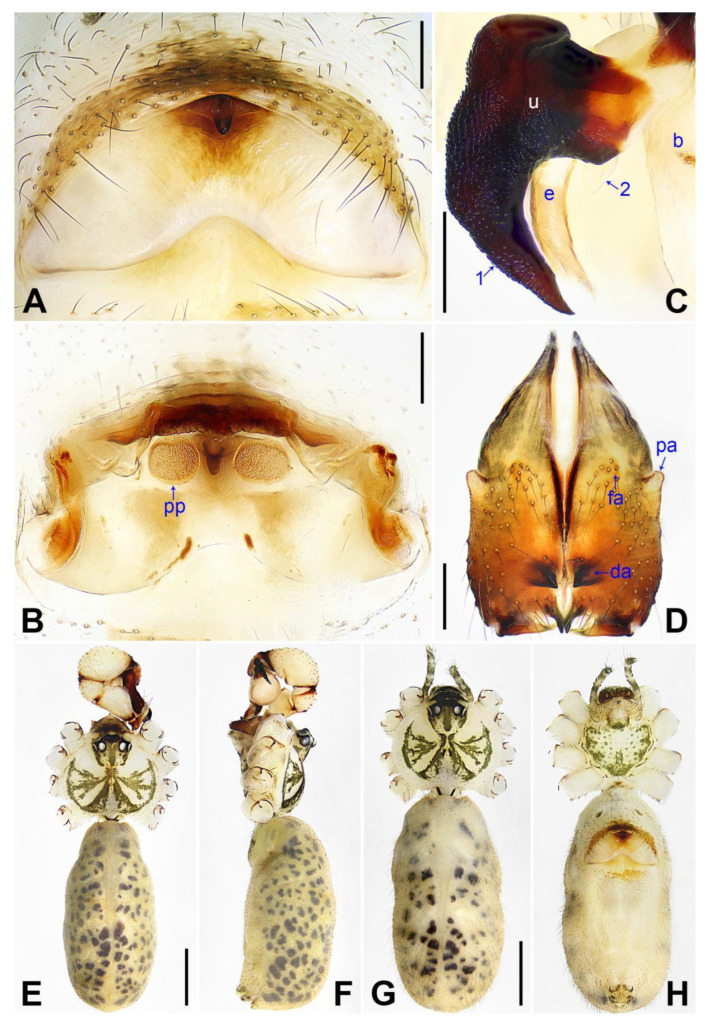
*Pholcus luliang* sp. nov., holotype male (**C**–**F**) and paratype female (**A**,**B**,**G**,**H**): (**A**) epigyne, ventral view; (**B**) vulva, dorsal view; (**C**) bulbal apophyses, prolateral view (arrow 1 points at distal apophysis, arrow 2 points at “pseudo-appendix”); (**D**) chelicerae, frontal view; (**E**,**G**) habitus, dorsal view; (**F**) habitus, lateral view; (**H**) habitus, ventral view. da = distal apophysis, e = embolus, fa = frontal apophysis, pa = proximo–lateral apophysis, pp = pore plate, u = uncus. Scale bars: (**A**–**D**) = 0.20 mm; (**E**–**H**) = 1.00 mm.

LSID: urn:lsid:zoobank.org:act:6D5551DC-FAA7-4443-8229-8ABC0EA57735

(Figure 9 and Figure 10).

**Holotype:** ♂ (SYNU-Ar00270), China, Shanxi, Lüliang, Jiaokou County, Shikou Town, Yunmengshan Scenic Spot, (36°54.13″ N, 111°6.45″ E, 1480 m), 4 August 2022, Zhi-Yuan Yao, Lan Yang & Lu-Dan Zhang leg.

**Paratypes:** 1♂ (SYNU-Ar00271), 3♀ (SYNU-Ar00272–Ar00274), same data as holotype.

**Etymology:** The specific name refers to the type locality and is a noun in apposition.

**Diagnosis:** The species resembles *P. zhongyang* sp. nov. in having similar male chelicerae and epigyne (Figure 18A,D) but can be distinguished by a procursus with a spine-shaped distal apophysis (arrow 2 in Figure 9C; slightly sclerotized, pointed distal apophysis in *P. zhongyang* sp. nov., arrow 2 in Figure 17C) and without a sclerotized ventro–distal apophysis (Figure 9B,C; present in *P. zhongyang* sp. nov., arrow in Figure 17B, arrow 4 in Figure 17C), by an uncus with a slightly curved distal apophysis (arrow 1 in Figure 10C; strongly curved in *P. zhongyang* sp. nov., arrow 1 in Figure 18C), and by almost round vulval pore plates (Figure 10B; nearly elliptic and anteriorly wide and posteriorly narrow in *P. zhongyang* sp. nov., Figure 18B).

**Description of holotype:** Male (SYNU-Ar00270). Total length 5.01 (5.26 with clypeus), carapace 1.55 long, 1.50 wide, opisthosoma 3.46 long, 1.72 wide. Leg I: 35.87 (9.05, 0.76, 9.50, 14.62, 1.94), leg II: 26.77 (7.37, 0.75, 6.65, 10.45, 1.55), leg III: 18.59 (5.64, 0.67, 4.05, 7.31, 0.92), leg IV: 24.43 (7.05, 0.66, 6.20, 9.17, 1.35); tibia I L/d: 63. Eye interdistances and diameters: PME–PME 0.23, PME 0.16, PME–ALE 0.04, AME–AME 0.05, AME 0.10. Sternum width/length: 1.24/1.02. Habitus as in Figure 10E,F. Carapace yellowish, with brown radiating marks and marginal brown bands; ocular area yellowish, with median and lateral brown bands; clypeus and sternum yellowish, with brown marks. Legs yellowish, but dark brown on patellae and whitish on distal parts of femora and tibiae, with darker rings on subdistal parts of femora and proximal and subdistal parts of tibiae. Opisthosoma yellowish, with dorsal and lateral spots. Clypeus with small frontal apophysis (Figure 10E). Chelicerae (Figure 10D) with pair of proximo–lateral apophyses, pair of distal apophyses with two teeth each, and pair of frontal apophyses. Pedipalp as in Figure 9A,B; trochanter with long (longer than wide), retrolaterally strongly bulged ventral apophysis; femur with small retrolatero–proximal apophysis and indistinct ventral protuberance; tibia with prolatero–ventral projection; procursus simple proximally but complex distally, with curved prolateral membranous process with sclerotized edge (arrow 1 in Figure 9C), spine-shaped distal apophysis (arrow 2 in Figure 9C), dorsal membranous lamella (arrow 3 in Figure 9C), and one strong and one slender dorsal spine (arrows in Figure 9D); uncus with curved, pointed distal apophysis (arrow 1 in Figure 10C) and scales; “pseudo-appendix” semi-transparent (arrow 2 in Figure 10C); embolus weakly sclerotized, with some transparent distal projections (Figure 10C). Retrolateral trichobothrium of tibia I at 5% proximally; legs with short vertical setae on tibiae, metatarsi, and tarsi; tarsus I with 30 distinct pseudosegments.

**Description of paratype:** Female (SYNU-Ar00272). Similar to male, habitus as in Figure 10G,H. Total length 4.86 (5.06 with clypeus), carapace 1.58 long, 1.72 wide, opisthosoma 3.28 long, 1.84 wide; tibia I: 6.67; tibia I L/d: 43. Eye interdistances and diameters: PME–PME 0.20, PME 0.14, PME–ALE 0.06, AME–AME 0.05, AME 0.10. Sternum width/length: 1.26/0.95. Clypeus brown, without frontal apophysis. Epigyne (Figure 10A) postero–medially strongly curved, with median brown marks and knob. Vulva (Figure 10B) with curved, posteriorly sclerotized anterior arch, pair of nearly round pore plates, and pair of posterior sclerites.

**Variation:** Tibia I in paratype male (SYNU-Ar00271): 10.13. Tibia I in the other two paratype females (SYNU-Ar00273, Ar00274): 7.05, 7.40.

**Natural history:** The species was found on rock walls.

**Distribution:** China (Shanxi, type locality; Figure 1).

***Pholcus luya* Peng & Zhang, 2013**

*Pholcus luya* Peng & Zhang, 2013: 77, Figures 3A–G, 4A–F [15]. Lu et al., 2022: S24, Figure S26A–D [17].

**Material examined:** 1♂ (SYNU-Ar00275), 2♀ (SYNU-Ar00276, Ar00277), China, Shanxi, Lüliang, Lan County, Bailong Mountain Scenic Spot (38°19.05’ N, 111°28.23’ E, 1653 m), 7 August 2022, Zhi-Yuan Yao, Lan Yang & Lu-Dan Zhang leg.

**Diagnosis and description:** See Peng & Zhang [15] and Lu et al. [17].

**Natural history:** The species was found on rock walls.

**Distribution:** China (Shanxi, Figure 1).

***Pholcus wenshui* Zhao, Li & Yao, sp. nov.**

**Figure 11:**
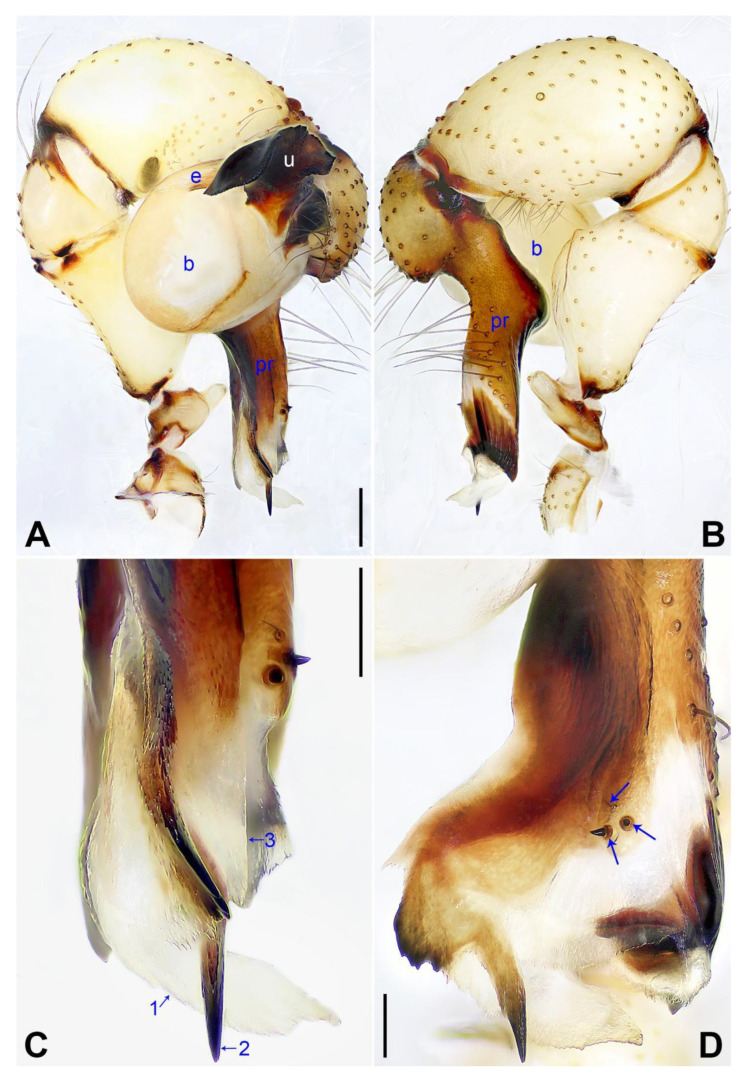
*Pholcus wenshui* sp. nov., holotype male: (**A**) pedipalp, prolateral view; (**B**) pedipalp, retrolateral view; (**C**) distal part of procursus, prolateral view (arrow 1 points at prolateral membranous process, arrow 2 points at spine-shaped distal apophysis, arrow 3 points at dorsal membranous lamella); (**D**) distal part of procursus, dorsal view (arrows point at dorsal spines). b = bulb, e = embolus, pr = procursus, u = uncus. Scale bars: (**A**,**B**) = 0.20 mm; (**C**,**D**) = 0.10 mm.

**Figure 12:**
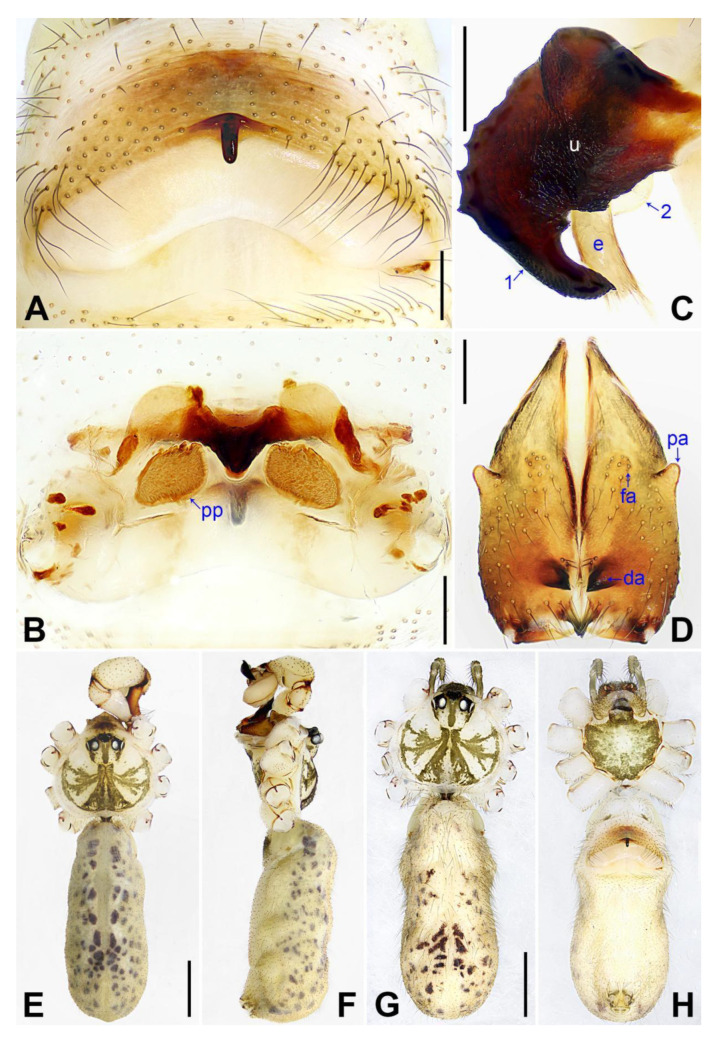
*Pholcus wenshui* sp. nov., holotype male (**C**–**F**) and paratype female (**A**,**B**,**G**,**H**): (**A**) epigyne, ventral view; (**B**) vulva, dorsal view; (**C**) bulbal apophyses, prolateral view (arrow 1 points at wide distal apophysis, arrow 2 points at ‘pseudo-appendix’); (**D**) chelicerae, frontal view; (**E**), (**G**) habitus, dorsal view; (**F**) habitus, lateral view; (**H**) habitus, ventral view. da = distal apophysis, e = embolus, fa = frontal apophysis, pa = proximo–lateral apophysis, pp = pore plate, u = uncus. Scale bars: (**A**–**D**) = 0.20 mm; (**E**–**H**) = 1.00 mm.

LSID: urn:lsid:zoobank.org:act:4FBD68DB-AE70-4EAF-A795-6DC116BA57B1

(Figure 11 and Figure 12).

**Holotype:** ♂ (SYNU-Ar00278), China, Shanxi, Lüliang, Wenshui County, roadside of Guwu Road (37°32.02’ N, 111°38.85’ E, 1468 m), 5 August 2022, Zhi-Yuan Yao, Lan Yang & Lu-Dan Zhang leg.

**Paratypes:** 2♂ (SYNU-Ar00279, Ar00270), 3♀ (SYNU-Ar00281–Ar00283), same data as holotype.

**Etymology:** The specific name refers to the type locality and is a noun in apposition.

**Diagnosis:** The species resembles *P. jiaocheng* sp. nov. in having similar male chelicerae and vulva (Figure 4B,D) but can be distinguished by a prolateral membranous process of the procursus with a strongly sclerotized edge (arrow 1 in Figure 11C; slightly sclerotized edge in *P. jiaocheng* sp. nov., arrow 1 in Figure 3C), by a procursus with a spine-shaped distal apophysis (arrow 2 in Figure 11C; slightly sclerotized, pointed distal apophysis in *P. jiaocheng* sp. nov., arrow 2 in Figure 3C), by an uncus with a wide distal apophysis and sawtoothed edge (arrow 1 in Figure 12C; slender distal apophysis in *P. jiaocheng* sp. nov., arrow 1 in Figure 4C), and by an epigynal plate strongly curved in the ventral view (Figure 12A; slightly curved in *P. jiaocheng* sp. nov., Figure 4A).

**Description of holotype:** Male (SYNU-Ar00278). Total length 5.23 (5.51 with clypeus), carapace 1.58 long, 1.56 wide, opisthosoma 3.65 long, 1.53 wide. Leg I: 38.77 (9.94, 0.75, 10.13, 15.90, 2.05), leg II: 28.06 (7.69, 0.68, 6.98, 11.09, 1.62), leg III: 19.78 (5.64, 0.63, 4.86, 7.45, 1.20), leg IV: 25.83 (7.25, 0.66, 6.53, 9.94, 1.45); tibia I L/d: 63. Eye interdistances and diameters: PME–PME 0.24, PME 0.16, PME–ALE 0.05, AME–AME 0.05, AME 0.11. Sternum width/length: 1.23/1.01. Habitus as in Figure 12E,F. Carapace yellowish, with brown radiating marks and marginal brown bands; ocular area yellowish, with median and lateral brown bands; clypeus and sternum yellowish, with brown marks. Legs yellowish, but dark brown on patellae and whitish on distal parts of femora and tibiae, with darker rings on subdistal parts of femora and proximal and subdistal parts of tibiae. Opisthosoma yellowish, with dorsal and lateral spots. Clypeus with small frontal apophysis (Figure 12E). Chelicerae (Figure 12D) with pair of proximo–lateral apophyses, pair of distal apophyses with two teeth each, and pair of frontal apophyses. Pedipalp as in Figure 11A,B; trochanter with long (longer than wide), retrolaterally strongly bulged ventral apophysis; femur with small retrolatero–proximal apophysis and indistinct ventral protuberance; tibia with prolatero–ventral projection; procursus simple proximally but complex distally, with curved, marginally sclerotized prolateral membranous process (arrow 1 in Figure 11C), spine-shaped distal apophysis (arrow 2 in Figure 11C), dorsal membranous lamella (arrow 3 in Figure 11C), and two strong and one slender dorsal spines (arrowed in Figure 11D); uncus with wide, curved distal apophysis (arrow 1 in Figure 12C), sawtoothed edge, and scales; “pseudo-appendix” semi-transparent (arrow 2 in Figure 12C); embolus weakly sclerotized, with some transparent distal projections (Figure 12C). Retrolateral trichobothrium of tibia I at 5% proximally; legs with short vertical setae on tibiae, metatarsi, and tarsi; tarsus I with 35 distinct pseudosegments.

**Description of paratype:** Female (SYNU-Ar00281). Similar to male, habitus as in Figure 12G,H. Total length 5.00 (5.19 with clypeus), carapace 1.56 long, 1.60 wide, opisthosoma 3.44 long, 1.50 wide; tibia I: 8.01; tibia I L/d: 48. Eye interdistances and diameters: PME–PME 0.22, PME 0.17, PME–ALE 0.05, AME–AME 0.05, AME 0.11. Sternum width/length: 1.22/0.98. Clypeus brown, without frontal apophysis. Epigyne (Figure 12A) postero–medially strongly curved, with knob. Vulva (Figure 12B) with M-shaped, sclerotized anterior arch, pair of nearly elliptic pore plates, and pair of indistinct posterior sclerites.

**Variation:** Tibia I in two paratype males (SYNU-Ar00279, Ar00280): 10.12, 10.96. Tibia I in the other two paratype females (SYNU-Ar00282, Ar00283): 7.37, 8.20.

**Natural history:** The species was found on rock walls.

**Distribution:** China (Shanxi, type locality; Figure 1).

***Pholcus xiangfen* Zhao, Li & Yao, sp. nov.**

**Figure 13:**
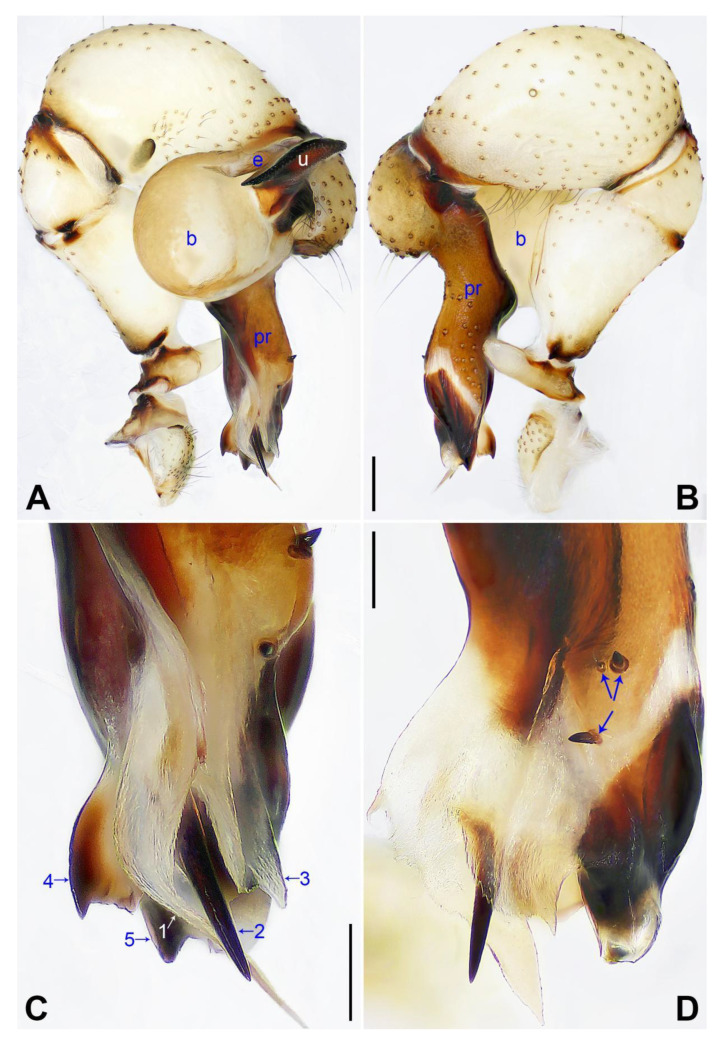
*Pholcus xiangfen* sp. nov., holotype male: (**A**) flipped right pedipalp, prolateral view; (**B**) flipped right pedipalp, retrolateral view; (**C**) distal part of flipped right procursus, prolateral view (arrow 1 points at prolateral membranous process, arrow 2 points at spine-shaped distal apophysis, arrow 3 points at dorsal membranous lamella, arrows 4, 5 point at ventro–subdistal and ventro–distal apophyses, respectively); (**D**) distal part of flipped right procursus, dorsal view (arrows point at dorsal spines). b = bulb, e = embolus, pr = procursus, u = uncus. Scale bars: (**A**,**B**) = 0.20 mm; (**C**,**D**) = 0.10 mm.

**Figure 14:**
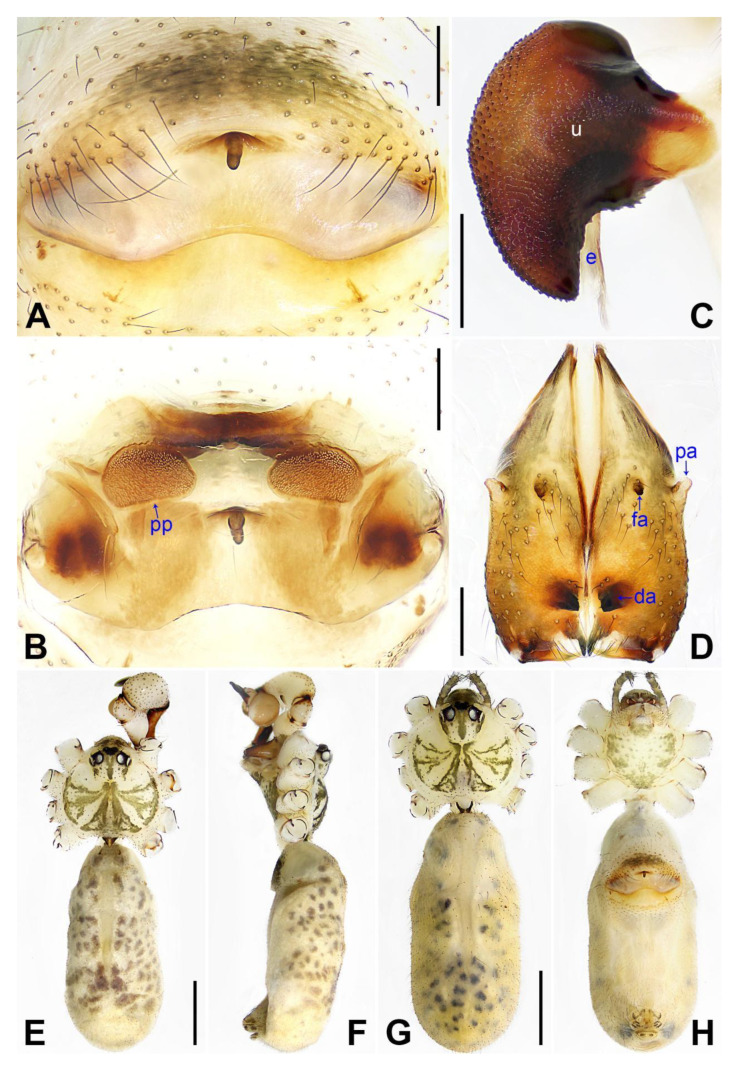
*Pholcus xiangfen* sp. nov., holotype male (**C**–**F**) and paratype female (**A**,**B**,**G**,**H**): (**A**) epigyne, ventral view; (**B**) vulva, dorsal view; (**C**) flipped right bulbal apophyses, prolateral view; (**D**) chelicerae, frontal view; (**E**), (**G**) habitus, dorsal view; (**F**) habitus, lateral view; (**H**) habitus, ventral view. da = distal apophysis, e = embolus, fa = frontal apophysis, pa = proximo–lateral apophysis, pp = pore plate, u = uncus. Scale bars: (**A**–**D**) = 0.20 mm; (**E**–**H**) = 1.00 mm.

LSID: urn:lsid:zoobank.org:act:DCE24ED6-BA8B-4473-A3C9-ABF066B945F1

(Figure 13 and Figure 14).

**Holotype:** ♂ (SYNU-Ar00284), China, Shanxi, Linfen, Xiangfen County, Xiangling Town, Huangya Village, Guye Mountain, near Yunwu Temple (36°8.72′ N, 111°21.45′ E, 808 m), 3 August 2022, Zhi-Yuan Yao, Lan Yang & Lu-Dan Zhang leg.

**Paratype:** 1♀ (SYNU-Ar00285), same data as holotype.

**Etymology:** The specific name refers to the type locality and is a noun in apposition.

**Diagnosis:** The species resembles *P. linfen* sp. nov. in having similar male chelicerae, uncus and epigyne (Figure 6A,C,D), but can be distinguished by the prolateral membranous process of the procursus without a curved sclerotized apophysis (arrow 1 in Figure 13C; present in *P. linfen* sp. nov., arrow 1 in Figure 5C), by a procursus with a spine-shaped distal apophysis (arrow 2 in Figure 13C; absent in *P. linfen* sp. nov., Figure 5C), by a procursus with sclerotized ventro–subdistal and ventro–distal apophyses (arrows 4, 5 in Figure 13C; with a slightly sclerotized ventro–distal apophysis in *P. linfen* sp. nov., arrow in Figure 5B, arrow 3 in Figure 5C), by nearly semi-circular vulval pore plates (Figure 14B; nearly trapezoidal in *P. linfen* sp. nov., Figure 6B), and by male clypeus without a small frontal apophysis (Figure 14E; present in *P. linfen* sp. nov., Figure 6E).

**Description of holotype:** Male (SYNU-Ar00284). Total length 4.82 (4.94 with clypeus), carapace 1.42 long, 1.69 wide, opisthosoma 3.40 long, 1.53 wide. Leg I: 42.44 (10.64, 0.71, 10.83, 17.56, 2.70), leg II: 29.00 (7.84, 0.69, 7.22, 11.47, 1.78), leg III: 19.93 (5.71, 0.63, 4.78, 7.63, 1.18), leg IV: 24.19 (5.45, 0.63, 6.60, 10.19, 1.32); tibia I L/d: 68. Eye interdistances and diameters: PME–PME 0.23, PME 0.14, PME–ALE 0.03, AME–AME 0.05, AME 0.11. Sternum width/length: 1.14/0.90. Habitus as in Figure 14E,F. Carapace yellowish, with brown radiating marks and marginal brown bands; ocular area yellowish, with median and lateral brown bands; clypeus and sternum yellowish, with brown marks. Legs yellowish, but dark brown on patellae and whitish on distal parts of femora and tibiae, with darker rings on subdistal parts of femora and proximal and subdistal parts of tibiae. Opisthosoma yellowish, with dorsal and lateral spots. Chelicerae (Figure 14D) with pair of proximo–lateral apophyses, pair of distal apophyses with two teeth each, and pair of frontal apophyses. Pedipalp as in Figure 13A,B; trochanter with long (longer than wide), retrolaterally strongly bulged ventral apophysis; femur with small retrolatero–proximal apophysis and indistinct ventral protuberance; tibia with prolatero–ventral projection; procursus simple proximally but complex distally, with curved prolateral membranous process (arrow 1 in Figure 13C), spine-shaped distal apophysis (arrow 2 in Figure 13C), dorsal membranous lamella (arrow 3 in Figure 13C), sclerotized ventro–subdistal and ventro–distal apophyses (arrows 4, 5 in Figure 13C), and two strong and one slender dorsal spines (arrows in Figure 13D); uncus semi-circular, with scales (Figure 14C); “pseudo-appendix” semi-transparent (invisible in Figure 14C); embolus weakly sclerotized, with some transparent distal projections (Figure 14C). Retrolateral trichobothrium of tibia I at 2% proximally; legs with short vertical setae on tibiae, metatarsi, and tarsi; tarsus I with 35 distinct pseudosegments.

**Description of paratype:** Female (SYNU-Ar00285). Similar to male, habitus as in Figure 14G,H. Total length 4.71 (4.90 with clypeus), carapace 1.39 long, 1.60 wide, opisthosoma 3.32 long, 1.48 wide; tibia I: 7.35; tibia I L/d: 53. Eye interdistances and diameters: PME–PME 0.20, PME 0.15, PME–ALE 0.03, AME–AME 0.05, AME 0.08. Sternum width/length: 1.11/0.85. Epigyne (Figure 14A) postero–medially curved, with knob. Vulva (Figure 14B) with slightly curved, medially sclerotized anterior arch, pair of nearly semi-circular pore plates, and pair of long lateral sclerites.

**Natural history:** The species was found on rock walls.

**Distribution:** China (Shanxi, type locality; Figure 1).

***Pholcus xuanzhong* Zhao, Li & Yao, sp. nov.**

**Figure 15:**
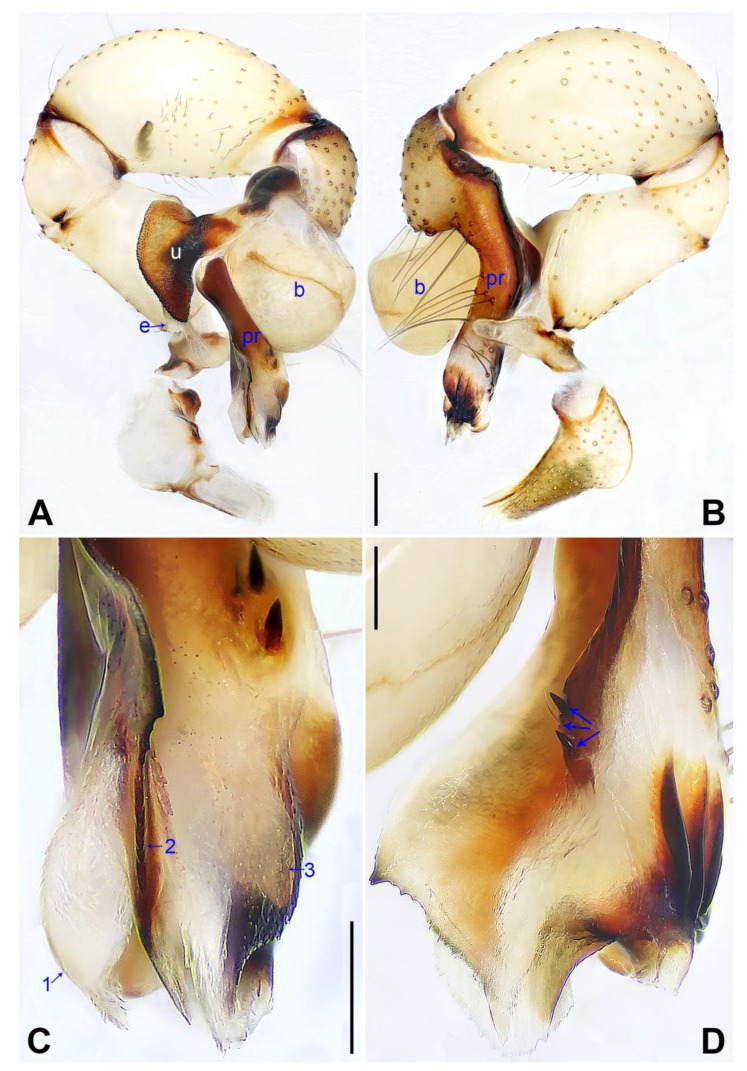
*Pholcus xuanzhong* sp. nov., holotype male: (**A**) pedipalp, prolateral view; (**B**) pedipalp, retrolateral view; (**C**) distal part of procursus, prolateral view (arrow 1 points at prolateral membranous process, arrow 2 points at prolateral sclerite, arrow 3 points at dorsal membranous lamella); (**D**) distal part of procursus, dorsal view (arrows point at dorsal spines). b = bulb, e = embolus, pr = procursus, u = uncus. Scale bars: (**A**,**B**) = 0.20 mm; (**C**,**D**) = 0.10 mm.

**Figure 16:**
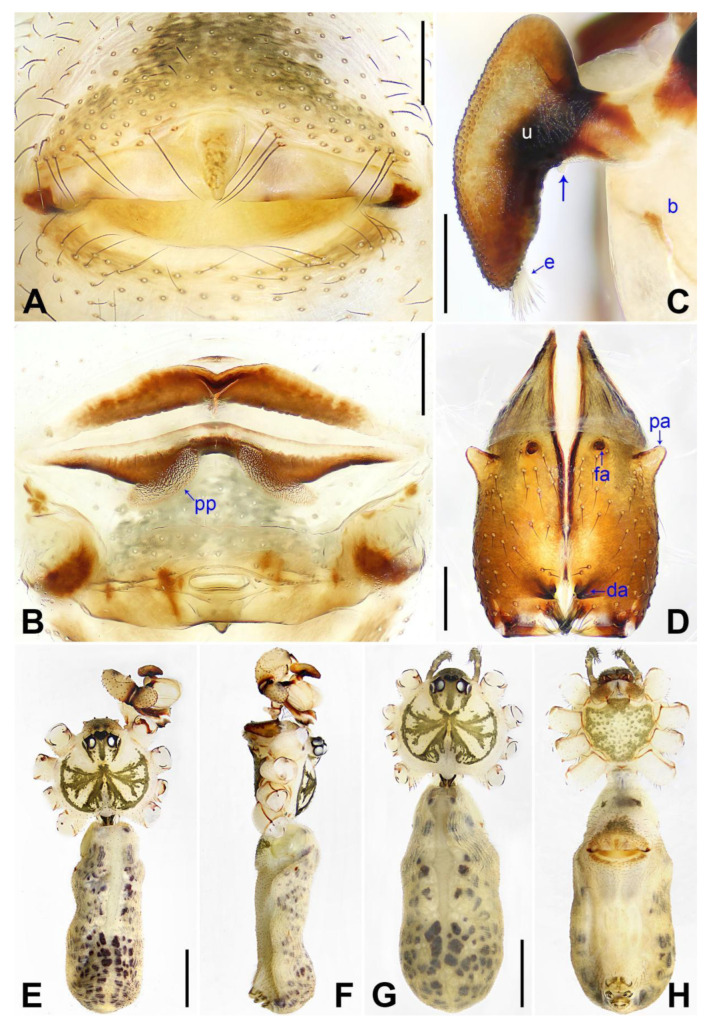
*Pholcus xuanzhong* sp. nov., holotype male (**C**–**F**) and paratype female (**A**,**B**,**G**,**H**): (**A**) epigyne, ventral view; (**B**) vulva, dorsal view; (**C**) bulbal apophyses, prolateral view (arrow points at ‘pseudo-appendix’); (**D**) chelicerae, frontal view; (**E**), (**G**) habitus, dorsal view; (**F**) habitus, lateral view; (**H**) habitus, ventral view. da = distal apophysis, e = embolus, fa = frontal apophysis, pa = proximo–lateral apophysis, pp = pore plate, u = uncus. Scale bars: (**A**–**D**) = 0.20 mm; (**E**–**H**) = 1.00 mm.

LSID: urn:lsid:zoobank.org:act:57C2E8BC-4D4C-49AE-BB3D-C0961DD2DBE1

(Figure 15 and Figure 16).

**Holotype:** ♂ (SYNU-Ar00286), China, Shanxi, Lüliang, Jiaocheng County, near Xuanzhong Temple, roadside of Y011 (37°33.33’ N, 112°5.00’ E, 910 m), 6 August 2022, Zhi-Yuan Yao, Lan Yang & Lu-Dan Zhang leg.

**Paratypes:** 1♂ (SYNU-Ar00287), 3♀ (SYNU-Ar00288–Ar00280), same data as holotype.

**Etymology:** The specific name refers to the type locality and is a noun in apposition.

**Diagnosis:** The species can be distinguished from all congeners from Lüliang Mountains by the procursus without spine-shaped distal apophysis or a slightly sclerotized, pointed distal apophysis (Figure 15C), by a nearly elliptic uncus (Figure 16C), by a nearly triangular epigynal plate, with wedge-shaped knob (Figure 16A), and by vulval pore plates long and curved (Figure 16B).

**Description of holotype:** Male (SYNU-Ar00286). Total length 4.95 (5.12 with clypeus), carapace 1.55 long, 1.85 wide, opisthosoma 3.40 long, 1.38 wide. Leg I: missing, femur II: 8.14 (other segments missing), leg III: missing, femur IV: 7.95 (other segments missing). Eye interdistances and diameters: PME–PME 0.21, PME 0.17, PME–ALE 0.06, AME–AME 0.04, AME 0.10. Sternum width/length: 1.21/1.05. Habitus as in Figure 16E,F. Carapace yellowish, with brown radiating marks and marginal brown bands; ocular area yellowish, with median and lateral brown bands; clypeus and sternum yellowish, with brown marks. Legs yellowish, but dark brown on patellae and whitish on distal parts of femora and tibiae, with darker rings on subdistal parts of femora and proximal and subdistal parts of tibiae. Opisthosoma yellowish, with dorsal and lateral spots. Chelicerae (Figure 16D) with pair of proximo–lateral apophyses, pair of distal apophyses with two teeth each, and pair of frontal apophyses. Pedipalp as in Figure 15A,B; trochanter with long (longer than wide), retrolaterally strongly bulged ventral apophysis; femur with small retrolatero–proximal apophysis and indistinct ventral protuberance; tibia with prolatero–ventral projection; procursus simple proximally but complex distally, with prolateral membranous process (arrow 1 in Figure 15C), prolateral sclerite (arrow 2 in Figure 15C), dorsal membranous lamella (arrow 3 in Figure 15C), and two strong and one slender dorsal spines (arrows in Figure 15D); uncus nearly elliptic, with scaly edge (Figure 16C); “pseudo-appendix” semi-transparent (arrow in Figure 16C); embolus weakly sclerotized, with some transparent distal projections (Figure 16C).

**Description of paratype:** Female (SYNU-Ar00288). Similar to male, habitus as in Figure 16G,H. Total length 5.00 (5.13 with clypeus), carapace 1.52 long, 1.70 wide, opisthosoma 3.48 long, 1.64 wide; Leg I: 32.38 (8.08, 0.64, 8.40, 13.21, 2.05); tibia I L/d: 53. 

Eye interdistances and diameters: PME–PME 0.18, PME 0.15, PME–ALE 0.06 AME–AME 0.05, AME 0.10. Sternum width/length: 1.12/0.86. Clypeus brown. Epigyne (Figure 16A) nearly triangular, with lateral brown marks and wedge-shaped knob. Vulva (Figure 16B) with nearly w-shaped anterior arch (curved sclerite anterior to arch) and pair of long curved pore plates. Retrolateral trichobothrium of tibia I at 3% proximally; legs with short vertical setae on tibiae, metatarsi, and tarsi; tarsus I with 35 distinct pseudosegments.

**Variation:** Tibia I in paratype male (SYNU-Ar00287): 12.05. Tibia I in the other two paratype females (SYNU-Ar00289, Ar00290): 8.46, 8.72.

**Natural history:** The species was found on rock walls.

**Distribution:** China (Shanxi, type locality; Figure 1).

***Pholcus zhongyang* Zhao, Li & Yao, sp. nov.**

**Figure 17:**
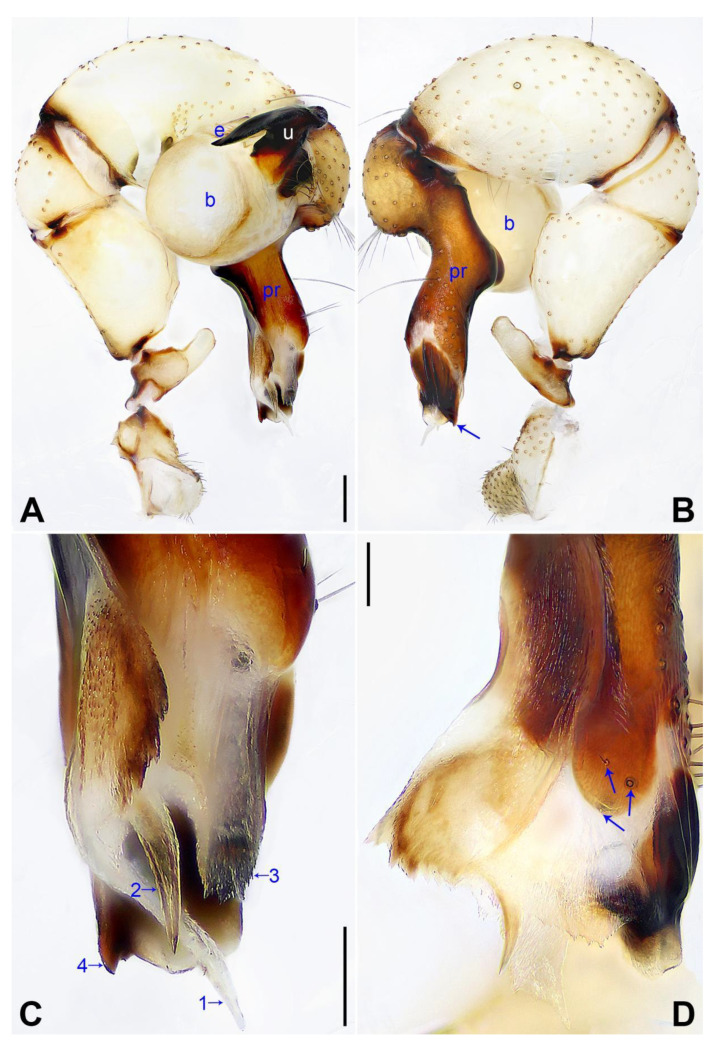
*Pholcus zhongyang* sp. nov., holotype male: (**A**) pedipalp, prolateral view; (**B**) pedipalp, retrolateral view (arrow points at ventro–distal apophysis); (**C**) distal part of procursus, prolateral view (arrow 1 points at prolateral membranous process, arrow 2 points at slightly sclerotized distal apophysis, arrow 3 points at dorsal membranous lamella, arrow 4 points at ventro–distal apophysis); (**D**) distal part of procursus, dorsal view (arrows point at dorsal spines). b = bulb, e = embolus, pr = procursus, u = uncus. Scale bars: (**A**,**B**) = 0.20 mm; (**C**,**D**) = 0.10 mm.

**Figure 18:**
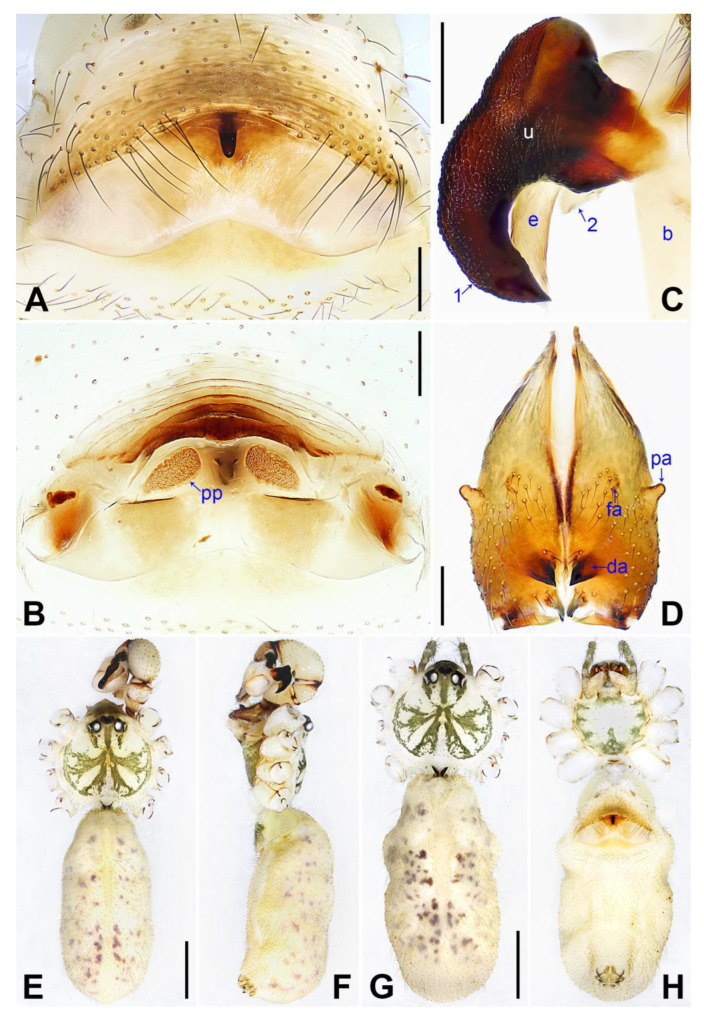
*Pholcus zhongyang* sp. nov., holotype male (**C**–**F**) and paratype female (**A**,**B**,**G**,**H**): (**A**) epigyne, ventral view; (**B**) vulva, dorsal view; (**C**) bulbal apophyses, prolateral view (arrow 1 points at distal apophysis, arrow 2 points at “pseudo-appendix”); (**D**) chelicerae, frontal view; (**E**,**G**) habitus, dorsal view; (**F**) habitus, lateral view; (**H**) habitus, ventral view. da = distal apophysis, e = embolus, fa = frontal apophysis, pa = proximo–lateral apophysis, pp = pore plate, u = uncus. Scale bars: (**A**–**D**) = 0.20 mm; (**E**–**H**) = 1.00 mm.

LSID: urn:lsid:zoobank.org:act:2BF7130E-1F88-4428-94A5-F3F4CD2CB3A6

(Figure 17 and Figure 18).

**Holotype:** ♂ (SYNU-Ar00291), China, Shanxi, Lüliang, Zhongyang County, Nuanquan Town, Xiahui Village, roadside of Subei Road (37°11.07’ N, 111°13.75’ E, 1310 m), 5 August 2022, Zhi-Yuan Yao, Lan Yang & Lu-Dan Zhang leg.

**Paratypes:** 2♂ (SYNU-Ar00292, Ar00293), 3♀ (SYNU-Ar00294–Ar00296), same data as holotype.

**Etymology:** The specific name refers to the type locality and is a noun in apposition.

**Diagnosis:** The species resembles *P. luliang* sp. nov. in having similar male chelicerae and epigyne (Figure 10A,D) but can be distinguished by a procursus with a slightly sclerotized, pointed distal apophysis (arrow 2 in Figure 17C; spine-shaped distal apophysis in *P. luliang* sp. nov., arrow 2 in Figure 9C) and sclerotized ventro–distal apophysis (arrow in Figure 17B, arrow 4 in Figure 17C; absent in *P. luliang* sp. nov., Figure 9B,C), by an uncus with a strongly curved distal apophysis (arrow 1 in Figure 18C; slightly curved in *P. luliang* sp. nov., arrow 1 in Figure 10C), and by vulval pore plates nearly elliptic and anteriorly wide and posteriorly narrow (Figure 18B; nearly round in *P. luliang* sp. nov., Figure 10B).

**Description of holotype:** Male (SYNU-Ar00291). Total length 5.14 (5.32 with clypeus), carapace 1.62 long, 1.80 wide, opisthosoma 3.52 long, 1.66 wide. Leg I: 36.76 (9.15, 0.74, 9.65, 15.19, 2.03), leg II: 24.78 (6.99, 0.68 6.32, 9.34, 1.45), leg III: 17.82 (5.25, 0.64, 4.50, 6.18, 1.25), leg IV: 24.29 (6.91, 0.65, 6.20, 9.15, 1.38); tibia I L/d: 64. Eye interdistances and diameters: PME–PME 0.26, PME 0.16, PME–ALE 0.04, AME–AME 0.04, AME 0.09. Sternum width/length: 1.18/1.03. Habitus as in Figure 18E,F. Carapace yellowish, with brown radiating marks and marginal brown bands; ocular area yellowish, with median and lateral brown bands; clypeus brown; sternum yellowish, with marginal brown marks. Legs yellowish, but dark brown on patellae and whitish on distal parts of femora and tibiae, with darker rings on subdistal parts of femora and proximal and subdistal parts of tibiae. Opisthosoma yellowish, with dorsal and lateral spots. Clypeus with small frontal apophysis (Figure 18E). Chelicerae (Figure 18D) with pair of proximo–lateral apophyses, pair of distal apophyses with two teeth each, and pair of frontal apophyses. Pedipalp as in Figure 17A,B; trochanter with long (longer than wide), retrolaterally strongly bulged ventral apophysis; femur with small retrolatero–proximal apophysis and indistinct ventral protuberance; tibia with prolatero–ventral projection; procursus simple proximally but complex distally, with curved prolateral membranous process with sclerotized edge (arrow 1 in Figure 17C), slightly sclerotized, pointed distal apophysis (arrow 2 in Figure 17C), dorsal membranous lamella (arrow 3 in Figure 17C), and two strong and one slender dorsal spines (arrows in Figure 17D); uncus with strongly curved, pointed distal apophysis (arrow 1 in Figure 18C) and scales; “pseudo-appendix” semi-transparent (arrow 2 in Figure 18C); embolus weakly sclerotized, with some transparent distal projections (Figure 18C). Retrolateral trichobothrium of tibia I at 3% proximally; legs with short vertical setae on tibiae, metatarsi, and tarsi; tarsus I with 33 distinct pseudosegments.

**Description of paratype:** Female (SYNU-Ar00294). Similar to male, habitus as in Figure 18G,H. Total length 4.94 (5.06 with clypeus), carapace 1.48 long, 1.80 wide, opisthosoma 3.46 long, 1.88 wide; tibia I: 7.76; tibia I L/d: 52. Eye interdistances and diameters: PME–PME 0.21, PME 0.15, PME–ALE 0.05, AME–AME 0.03, AME 0.08. Sternum width/length: 1.16/0.95. Clypeus without frontal apophysis. Epigyne (Figure 18A) postero–medially strongly curved, with median brown marks and knob. Vulva (Figure 18B) with curved, medially posteriorly sclerotized anterior arch, pair of nearly elliptic pore plates (anteriorly wide and posteriorly narrow), and pair of posterior sclerites.

**Variation:** Tibia I in two paratype males (SYNU-Ar00292, Ar00293): 9.61, 9.94. Tibia I in the other two paratype females (SYNU-Ar00295, Ar00296): 7.05, 7.82.

**Natural history:** The species was found on rock walls.

**Distribution:** China (Shanxi, type locality; Figure 1).

## Data Availability

Publication: LSID: urn:lsid:zoobank.org:pub:4CAA98A6-2CF3-44C8-94A5-4B363E23B603, *Pholcus jiaocheng* Zhao, Li & Yao, sp. nov.; LSID: urn:lsid:zoobank.org:act:B35A9380-213A-424B-AFDE-FE18A9B4B6F3, *Pholcus linfen* Zhao, Li & Yao, sp. nov.; LSID: urn:lsid:zoobank.org:act:057C81CA-8FF6-48CB-B07A-35FC81EEDACF, *Pholcus lishi* Zhao, Li & Yao, sp. nov.; LSID: urn:lsid:zoobank.org:act:8F8C4D58-9765-4471-AD6F-CA29C5EF41AC, *Pholcus luliang* Zhao, Li & Yao, sp. nov.; LSID: urn:lsid:zoobank.org:act:6D5551DC-FAA7-4443-8229-8ABC0EA57735, *Pholcus wenshui* Zhao, Li & Yao, sp. nov.; LSID: urn:lsid:zoobank.org:act:4FBD68DB-AE70-4EAF-A795-6DC116BA57B1, *Pholcus xiangfen* Zhao, Li & Yao, sp. nov.; LSID: urn:lsid:zoobank.org:act:DCE24ED6-BA8B-4473-A3C9-ABF066B945F1, *Pholcus xuanzhong* Zhao, Li & Yao, sp. nov.; LSID: urn:lsid:zoobank.org:act:57C2E8BC-4D4C-49AE-BB3D-C0961DD2DBE1, *Pholcus zhongyang* Zhao, Li & Yao, sp. nov.; LSID: urn:lsid:zoobank.org:act:2BF7130E-1F88-4428-94A5-F3F4CD2CB3A6. All data produced are available in this manuscript. The sequences are deposited in the GenBank under accession Nos. OQ706157–706196, OQ719631–719670, OQ719671–719710, OQ719758–719797.

## Supplementary Materials

The following supporting information can be downloaded at: https://www.mdpi.com/article/10.3390/insects14040364/s1, Figure S1: Phylogenetic tree of COI, derived from BI analysis; Figure S2: Results of bPTP and GMYC species delimitation analyses; Table S1: Primers used for amplification and sequencing; Table S2: Voucher specimen information. Refs. [43,44,45,46,47] cited in Supplementary Materials.
