# Supplementary material for: Diversity of Pholcus Spiders (Araneae: Pholcidae) in China’s Lüliang Mountains: An Integrated Morphological and Molecular Approach
Source: Insects. 2023 Apr 6;14(4):364. doi: 10.3390/insects14040364 (PMC10141095; doi:10.3390/insects14040364)
Supplement: Supplementary file 1 [file insects-14-00364-s001.zip › insects-2285321-supplementary.docx]

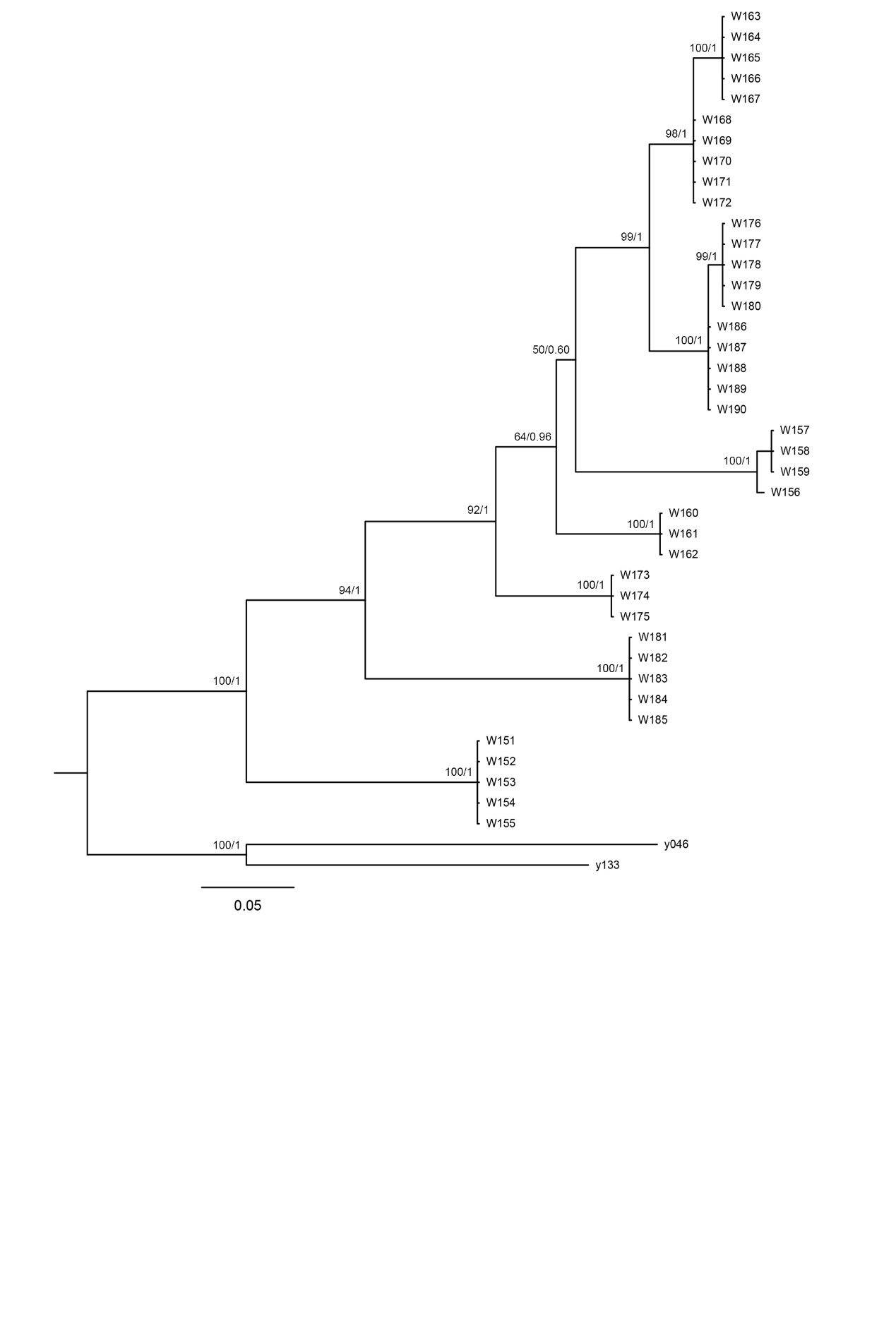


**Figure S1.** Phylogenetic tree of COI, derived from BI analysis. Boostrap values/Bayesian posterior probabilities are provided at the nodes.


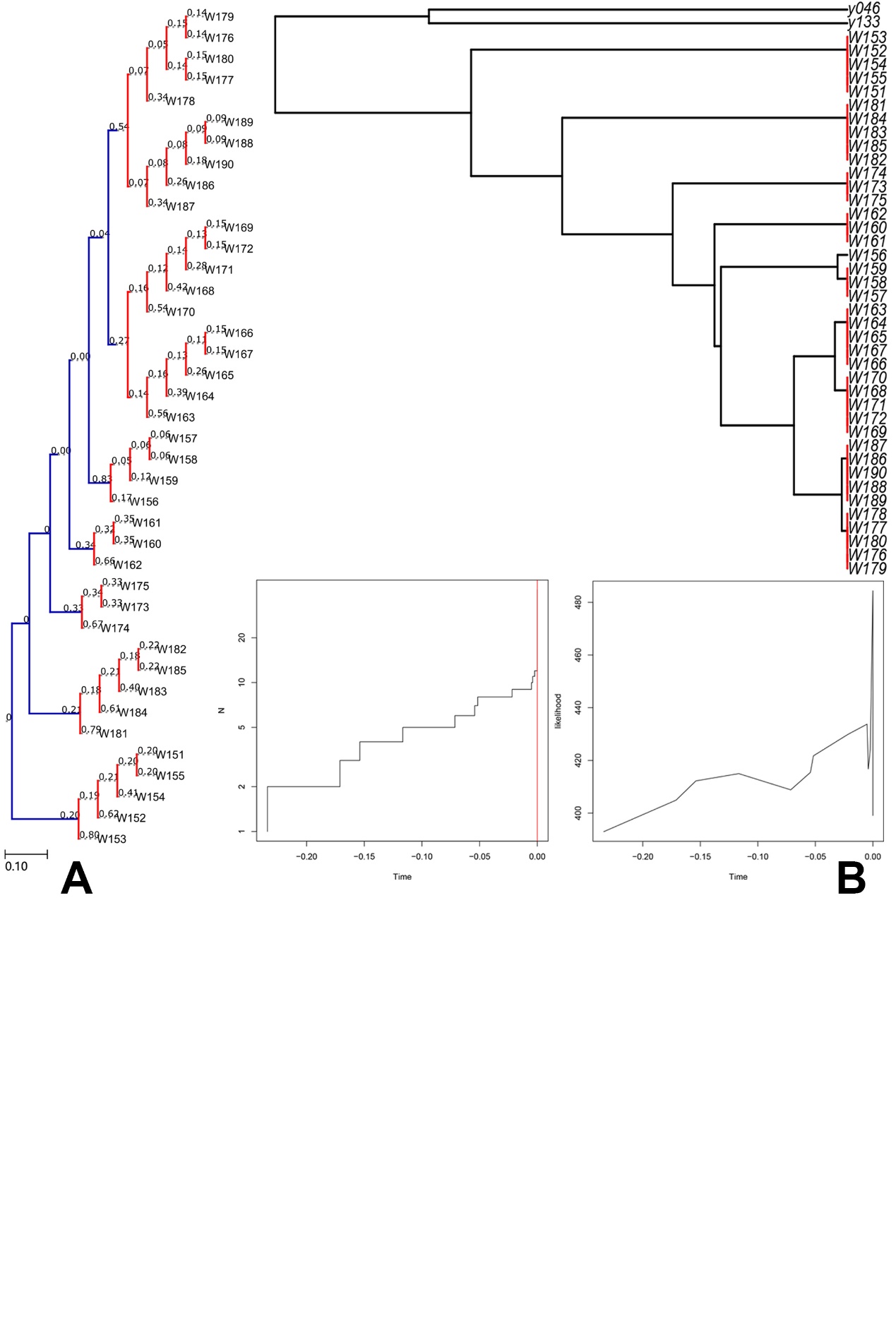


**Figure S2.** Results of bPTP and GMYC species delimitation analyses: (A) bPTP results based on the COI gene tree derived from the likelihood analysis; (B) GMYC results based on the COI gene tree, below are the lineages-through-time plot (left) and the single-threshold GMYC likelihood profile plot (right).

**Table S1.** Primers used for amplification and sequencing.

| Gene | Primer | F/R | Sequence 5'–3' | Reference |
| --- | --- | --- | --- | --- |
| COI | LCO1490  HCO2198 | F  R | GGTCAACAAATCATAAAGATATTGG  TAAACTTCAGGGTGACCAAAAAATCA | Folmer *et al*. [43]  Simon *et al*. [44] |
|  | COIJerry 2 | F | CAGCATTTGTTTTGATTTTTTGG |  |
|  | C1-N-2776 | R | GGATAATCAGAATATCGTCGAGG |  |
| H3 | H3af  H3ar | F  R | ATGGCTCGTACCAAGCAGACVGC  ATATCCTTRGGCATRATRGTGAC | Colgan *et al*. [45] |
| wnt | Spwgf1  Spwgr1  Wnt2f  Wnt2r | F  R  F  R | GYAAATGCCAYGGWATGTCMGG  ACTTGRCAACACCARTGAAAWG  CAGTGRAATGTRCARTTG  CNGTTCAAACTTGYTGGATG | [Blackledge *et* *al*.](#_ENREF_1) [46] |
| 28S | 28S rd4.8a | F | ACCTATTCTCAAACTTTAAATGG | Schwendinger & Giribet [47] |
|  | 28S rd7b1 | R | GACTTCCCTTACCTACAT |  |

F/R: Forward/Reverse PCR primer.

**Table S2.** Voucher specimen information.

| Species | Code | GenBank number | | | | Locality |
| --- | --- | --- | --- | --- | --- | --- |
|  |  | COI | H3 | wnt | 28S |  |
| *P*. *wenshui* sp. nov. | W176 | OQ706182 | OQ719656 | OQ719696 | OQ719783 | roadside of Guwu Road (37°32.02'N, 111°38.85'E, 1468 m), Wenshui County, Lüliang, Shanxi, China |
|  | W177 | OQ706183 | OQ719657 | OQ719697 | OQ719784 |  |
|  | W178 | OQ706184 | OQ719658 | OQ719698 | OQ719785 |  |
|  | W179 | OQ706185 | OQ719659 | OQ719699 | OQ719786 |  |
|  | W180 | OQ706186 | OQ719660 | OQ719700 | OQ719787 |  |
| *P*. *jiaocheng* sp. nov. | W186 | OQ706192 | OQ719666 | OQ719706 | OQ719793 | Badaogou Scenic Spot (37°50.97'N, 111°28.23'E, 1755 m), near Pangquangou Nature Reserve, Pangquangou Town, Jiaocheng County, Lüliang, Shanxi, China |
|  | W187 | OQ706193 | OQ719667 | OQ719707 | OQ719794 |  |
|  | W188 | OQ706194 | OQ719668 | OQ719708 | OQ719795 |  |
|  | W189 | OQ706195 | OQ719669 | OQ719709 | OQ719796 |  |
|  | W190 | OQ706196 | OQ719670 | OQ719710 | OQ719797 |  |
| *P*. *luliang* sp. nov. | W163 | OQ706169 | OQ719643 | OQ719683 | OQ719770 | Yunmengshan Scenic Spot (36°54.13''N, 111°6.45''E, 1480 m), Shikou Town, Jiaokou County, Lüliang, Shanxi, China |
|  | W164 | OQ706170 | OQ719644 | OQ719684 | OQ719771 |  |
|  | W165 | OQ706171 | OQ719645 | OQ719685 | OQ719772 |  |
|  | W166 | OQ706172 | OQ719646 | OQ719686 | OQ719773 |  |
|  | W167 | OQ706173 | OQ719647 | OQ719687 | OQ719774 |  |
| *P*. *zhongyang* sp. nov. | W168 | OQ706174 | OQ719648 | OQ719688 | OQ719775 | roadside of Subei Road (37°11.07'N, 111°13.75'E, 1310 m), Xiahui Village, Nuanquan Town, Zhongyang County, Lüliang, Shanxi, China |
|  | W169 | OQ706175 | OQ719649 | OQ719689 | OQ719776 |  |
|  | W170 | OQ706176 | OQ719650 | OQ719690 | OQ719777 |  |
|  | W171 | OQ706177 | OQ719651 | OQ719691 | OQ719778 |  |
|  | W172 | OQ706178 | OQ719652 | OQ719692 | OQ719779 |  |
| *P*. *linfen* sp. nov. | W156 | OQ706162 | OQ719636 | OQ719676 | OQ719763 | roadside of G309 (36°8.87'N, 111°0.58'E, 1292 m), Wangjiahe Village, Taitou Town, Ji County, Linfen, Shanxi, China |
|  | W157 | OQ706163 | OQ719637 | OQ719677 | OQ719764 |  |
|  | W158 | OQ706164 | OQ719638 | OQ719678 | OQ719765 |  |
|  | W159 | OQ706165 | OQ719639 | OQ719679 | OQ719766 |  |
| *P*. *xiangfen* sp. nov. | W160 | OQ706166 | OQ719640 | OQ719680 | OQ719767 | near Yunwu Temple (36°8.72'N, 111°21.45'E, 808 m), Guye Mountain, Huangya Village, Xiangling Town, Xiangfen County, Linfen, Shanxi, China |
|  | W161 | OQ706167 | OQ719641 | OQ719681 | OQ719768 |  |
|  | W162 | OQ706168 | OQ719642 | OQ719682 | OQ719769 |  |
| *P*. *lishi* sp. nov. | W173 | OQ706179 | OQ719653 | OQ719693 | OQ719780 | roadside of Y004 (37°30.17'N, 111°4.28'E, 908 m), near Anguo Temple, Wuya Mountain, Lishi District, Lüliang, Shanxi, China |
|  | W174 | OQ706180 | OQ719654 | OQ719694 | OQ719781 |  |
|  | W175 | OQ706181 | OQ719655 | OQ719695 | OQ719782 |  |
| *P*. *xuanzhong* sp. nov. | W181 | OQ706187 | OQ719661 | OQ719701 | OQ719788 | roadside of Y011 (37°33.33'N, 112°5.00'E, 910 m), near Xuanzhong Temple, Jiaocheng County, Lüliang, Shanxi, China |
|  | W182 | OQ706188 | OQ719662 | OQ719702 | OQ719789 |  |
|  | W183 | OQ706189 | OQ719663 | OQ719703 | OQ719790 |  |
|  | W184 | OQ706190 | OQ719664 | OQ719704 | OQ719791 |  |
|  | W185 | OQ706191 | OQ719665 | OQ719705 | OQ719792 |  |
| *P*. *luya* | W151 | OQ706157 | OQ719631 | OQ719671 | OQ719758 | Bailong Mountain Scenic Spot (38°19.05'N, 111°28.23'E, 1653 m), Lan County, Lüliang, Shanxi, China |
|  | W152 | OQ706158 | OQ719632 | OQ719672 | OQ719759 |  |
|  | W153 | OQ706159 | OQ719633 | OQ719673 | OQ719760 |  |
|  | W154 | OQ706160 | OQ719634 | OQ719674 | OQ719761 |  |
|  | W155 | OQ706161 | OQ719635 | OQ719675 | OQ719762 |  |
| *P. paralinzhou* | y046 | MW721825 | ON375203 | ON375294 | OQ721916 | Yuntaishan Scenic Spot, Xiuwu County, Jiaozuo, Henan, China |
| *P. taishan* | y133 | MW721826 | ON375204 | ON375293 | OQ721917 | Taishan Mountain, Taian, Shandong, China |
